# Tsukushi is a novel prognostic biomarker and correlates with tumor-infiltrating B cells in non-small cell lung cancer

**DOI:** 10.18632/aging.202403

**Published:** 2021-01-10

**Authors:** Hao Huang, Ding Zhang, Jinming Fu, Liyuan Zhao, Dapeng Li, Hongru Sun, Xinyan Liu, Jing Xu, Tian Tian, Lei Zhang, Ying Liu, Yuanyuan Zhang, Yashuang Zhao

**Affiliations:** 1 Department of Epidemiology, Public Health College, Harbin Medical University, Harbin, Heilongjiang Province, People’s Republic of China

**Keywords:** *TSKU*, tumor immune infiltration, prognosis, methylation, lung cancer

## Abstract

A recent study has reported that tsukushi (*TSKU*) may be related to the development of lung cancer. However, few studies focused on if *TSKU* associated with the prognosis and immune infiltration cells in non-small cell lung cancer (NSCLC). The effect of *TSKU* expression on prognosis with NSCLC was analyzed in the PrognoScan database and validated in The Cancer Genome Atlas. The composition of tumor infiltrating cells was quantified by methylation and expression data. We combined levels of tumor infiltrating cells with *TSKU* to evaluate the survival of patients. The analysis of a cohort (GSE31210, N=204) of lung cancer patients demonstrated that high *TSKU* expression was strongly associated with poor overall survival (*P* =1.90E-05). The combination of high *TSKU* expression and low infiltration B cells identified a subtype of patients with poor survival in NSCLC. Besides, the proportion of B cells in NSCLC patients with *TSKU* hypermethylation were higher than those patients with *TSKU* hypomethylation (*P* <0.001). Overall, high *TSKU* expression combined with low infiltration of B cells may associate with a poor prognosis of NSCLC patients. *TSKU* might be a potential prognostic biomarker involved in tumor immune infiltration in NSCLC.

## INTRODUCTION

Non-small cell lung cancer (NSCLC) is an extremely common and complicated malignant tumor worldwide [1]. Although NSCLC therapy has made significant progress recently, the 5-year overall survival (OS) rates remain low, at only approximately 25% [2–4]. Recently, immunotherapy was developed as a promising treatment for many cancers, including NSCLC. Studies found that tumor-infiltrating lymphocytes (TILs), such as CD8^+^ T cells and CD3^+^ T cells, up-regulated the expression of the markers of immunomodulator, which may affect the efficacy of immunotherapy and associate with a poor prognosis in NSCLC [5, 6]. DNA methylation plays a critical role in cell lineage specification [7, 8], and studies have indicated that DNA methylation can accurately estimate the distribution of cell subtypes in the blood [9, 10]. Therefore, DNA methylation may identify a specific molecular marker for the typing of immune cell subtypes, but it has rarely been explored in evaluating TILs in tumor tissue. In 2017, Jeschke, et al. first identified a methylation of TIL (MeTIL) signature by utilizing genome-wide DNA methylation profiling and then transformed the individual methylation values of the MeTIL markers into a score (MeTIL score) for the evaluation of TIL distributions to predict prognosis for breast cancer patients [11, 12]. Therefore, it is significant and imperative to uncover whether individual genes and their methylation statuses relate to TILs in tissue and prognosis in NSCLC.

Tsukushi (*TSKU*) is a protein-encoding gene that is a new member of the small leucine-rich repeat proteoglycan (SLRP) family. Previous studies have found that *Tsku* is involved in multiple cell signaling pathways, including the BMP, FGF, TGF-β, and Wnt pathways [13–15], and serves as a principal coordinator by interacting with signaling molecules in different animal tissues [16]. However, there have been few reports on exploring the functional significance of *TSKU* in human cancers. In March of 2019, the study published by Yamada, et al. first reported that *TSKU* overexpression enhanced cell proliferation activity and inhibited the epithelial-mesenchymal transition (EMT) in lung cancer cell lines [17]. Despite the possible functional potential of *TSKU* in cancer, little is known about whether *TSKU* is associated with clinical prognosis and tumor-infiltrating immune cells (TIICs) in human cancer. Previous studies have reported that TSKU serves as a modulator involved in the wound healing process via inhibition of TGF-β secretion from macrophages [18, 19]. Moreover, TGF-β is recognized as a pleiotropic cytokine with immunoregulatory properties that activate the differentiation and proliferation of immune cells, including T regulatory cells (Tregs) and T helper 17 (Th17) cells [20, 21]. Given the role of TSKU in regulating the expression of cytokines involved in immunoregulation in the wound healing process, we hypothesized that *TSKU* may be involved in the tumor immune response and have effects on prognosis in NSCLC.

Therefore, in this study, we analyzed the association between *TSKU* expression and the prognosis of NSCLC patients. We also evaluated the correlation of *TSKU* expression with TIIC levels in diverse tumor types. We further explored the relationship between *TSKU* methylation and the proportion of TIICs in lung cancer.

## RESULTS

### The expression levels of *TSKU* in different cancers

**Figure 1:**
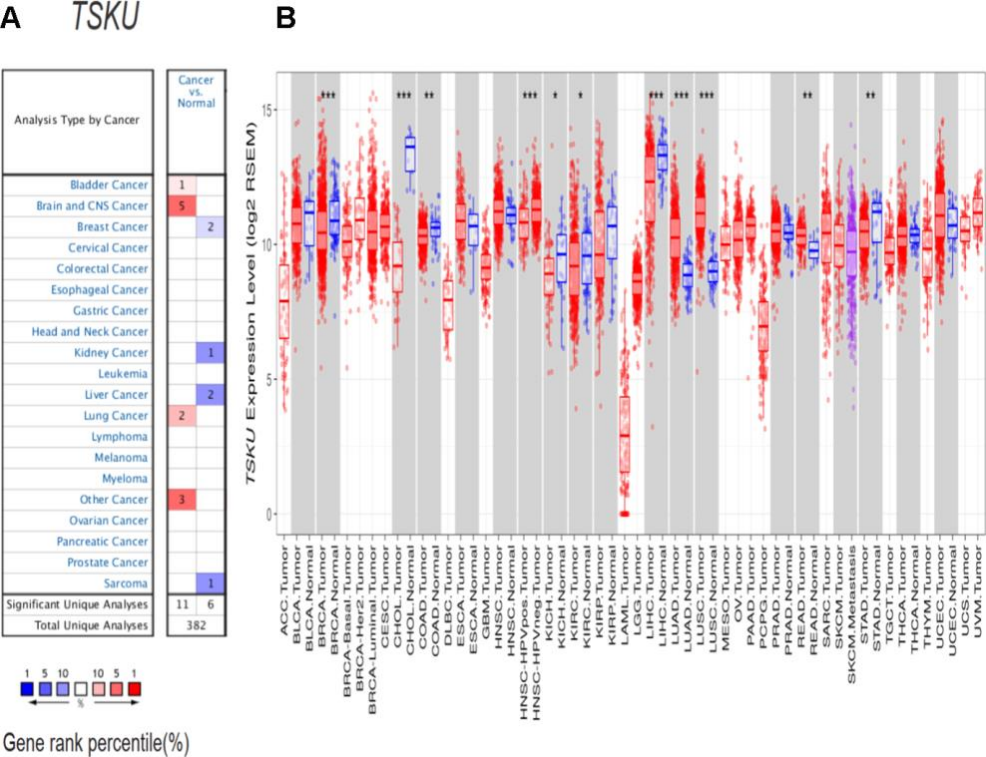
***TSKU* expression levels in different cancer types.** (**A**) Elevated or decreased *TSKU* expression in data sets of different cancers compared with normal tissues in the Oncomine database. (**B**) *TSKU* mRNA levels in multiple tumor types from the TCGA database were analyzed by TIMER. (*P < 0.05, **P <0.01, ***P < 0.001).

Based on the analysis of the Oncomine database, *TSKU* expression was higher in lung, bladder, brain and CNS, and other cancers than in normal tissues (Figure 1A). Lower expression of *TSKU* in tumors than in normal tissues was observed in breast, kidney, and liver cancers and sarcoma. The detailed results of *TSKU* expression in multiple cancer types are summarized in Supplementary Table 1.

To further validate the differential *TSKU* expression between different tumor and normal tissues, we analyzed TCGA (The Cancer Genome Atlas) data via the TIMER (Tumor Immune Estimation Resource) database. The expression of *TSKU* was significantly higher in LUAD (lung adenocarcinoma), LUSC (lung squamous cell carcinoma), and READ (rectum adenocarcinoma) datasets than in normal tissues (Figure 1B), while the expression of *TSKU* was lower in cancer than in normal tissues in BRCA (breast invasive carcinoma), CHOL (cholangiocarcinoma), COAD (colon adenocarcinoma), KICH (kidney chromophobe), KIRC (kidney renal clear cell carcinoma), LIHC (liver hepatocellular carcinoma), and STAD (stomach adenocarcinoma) datasets. These two databases showed consistent results in the differential *TSKU* expression between tumor and normal tissues in the lung cancer (LUAD and LUSC), BRCA, KICH, KIRC, and LIHC datasets.

### Associations between *TSKU* expression and prognosis in different cancers

**Figure 2:**
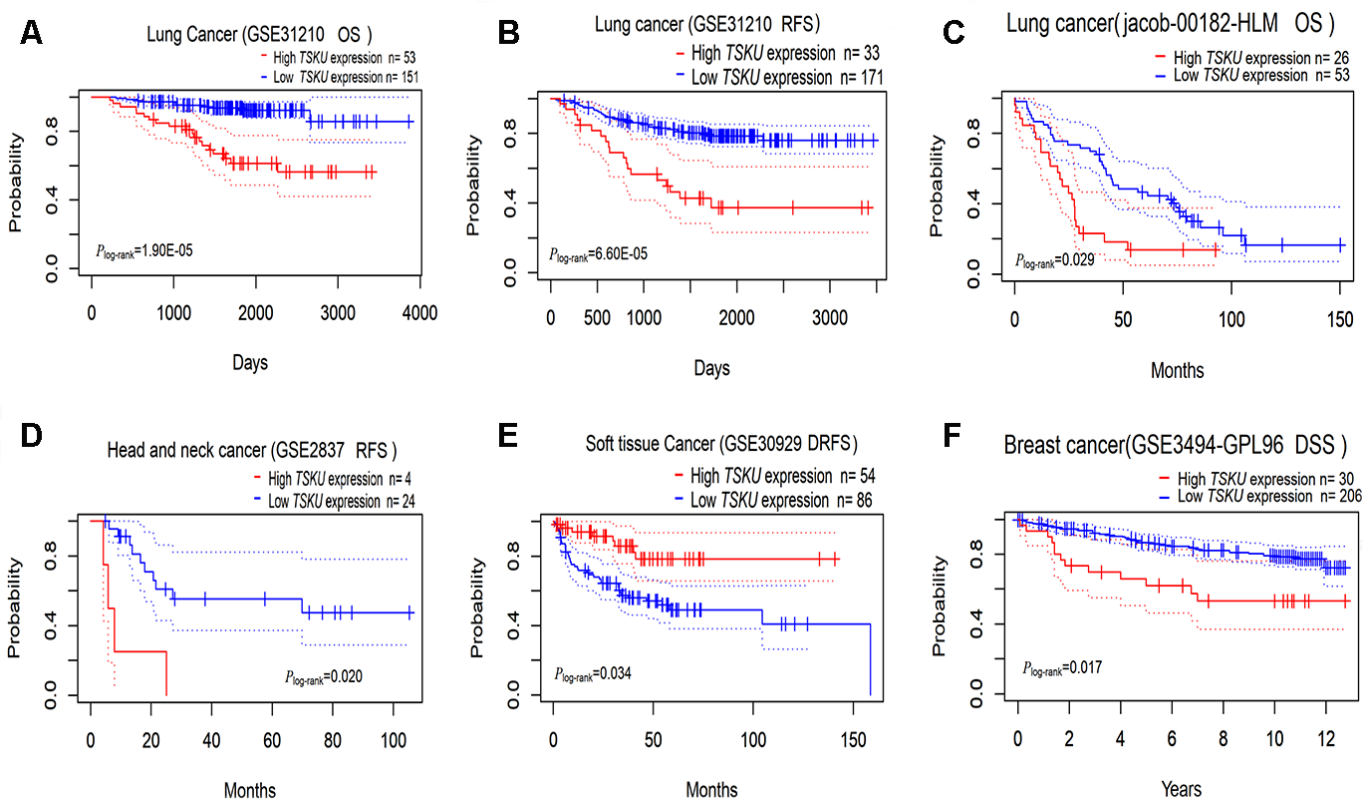
**Kaplan-Meier survival curves comparing high and low *TSKU* expression levels in different tumors via PrognoScan.** (**A**, **B**) Survival curves of OS and RFS in the lung cancer cohort (GSE31210, N =204); (**C**) Survival curves of OS in the lung cancer cohort (jacob-00182-HLM, N = 79); (**D**) Survival curves of RFS in the head and neck cancer cohort (GSE2837, N = 28); (**E**) Survival curves of DRFS in the soft tissue cancer cohort (GSE30929, N = 140); (**F**) Survival curves of DSS in the breast cancer cohort (GSE3494-GPL96, N = 236) OS, overall survival; DSS, disease Specific Survival; RFS, relapse-free survival; DRFS, distant recurrence-free survival.

We evaluated the impact of *TSKU* expression on the prognosis of various cancers using PrognoScan (Supplementary Table 2). *TSKU* expression has been significantly associated with the prognosis in some kinds of cancers, including lung, head and neck, breast, and soft tissue cancers (Figure 2A–2F). The cohort (GSE31210, N=204) of lung cancer patients in PrognoScan demonstrated Kaplan-Meier survival curves that showed patients in the high *TSKU* expression have poorer survival than those in low *TSKU* expression in overall survival (*P* =1.90E-05) and relapse-free survival (*P* =6.60E-05). High *TSKU* expression was strongly associated with poor overall survival of patients with lung cancer by multivariate Cox regression analysis, with HR_OS_ of 4.700 (95 % CI 2.360–9.360, *P* =1.10E-05) and HR_RFS_ of 3.400 (95 % CI 2.030–5.810, *P* =4.00E-06), respectively. In addition, the cohort (jacob-00182-HLM, N=79) of lung cancer patients with the high *TSKU* expression also showed poorer OS than those with low *TSKU* expression (*P*=0.029). Since the sample size is small for each cancer in PrognoScan, we merged GSE datasets in different survival statuses for every cancer type to perform a meta-analysis. The results of 14 types of meta-analysis included datasets in OS for 7 types of cancer, DFS (disease-free survival) for 2 types of cancer, DSS (disease specific survival) for 2 types of cancer, RFS (relapse-free survival) for 2 types of cancer, and DMFS (distant metastasis free survival) for 1 type of cancer. Among the 14 types of combination meta-analysis, we found that high *TSKU* expression was significantly associated with poorer OS in lung cancer and poorer DFS in colorectal cancer. (Lung cancer, N=1303, HR=1.260, 95% CI, 1.110-1.420; Colorectal cancer, N=413, HR=1.810, 95% CI, 1.000-3.290) (Supplementary Figure 1).

By further validating the association between *TSKU* expression and prognosis as determined by OS and DFS in 33 types of cancers from TCGA data via GEPIA (Gene Expression Profiling Interactive Analysis) (Supplementary Figure 2), we found that patients in the high *TSKU* expression showed poorer survival than those in the low *TSKU* expression in LUAD (*P*=0.004), ACC (adrenocortical carcinoma), KIRC, MESO (mesothelioma), PAAD (pancreatic adenocarcinoma), and THCA (thyroid carcinoma). However, patients in the low *TSKU* expression demonstrated poorer survival than those in the high *TSKU* expression in DLBC (lymphoid neoplasm diffuse large B-cell lymphoma), PRAD (prostate adenocarcinoma), and UVM (uveal melanoma). These two databases revealed that *TSKU* expression has an impact on the prognosis of some cancers, including lung cancer (LUAD).

### The correlation of *TSKU* expression with immune infiltration level in NSCLC

**Figure 3:**
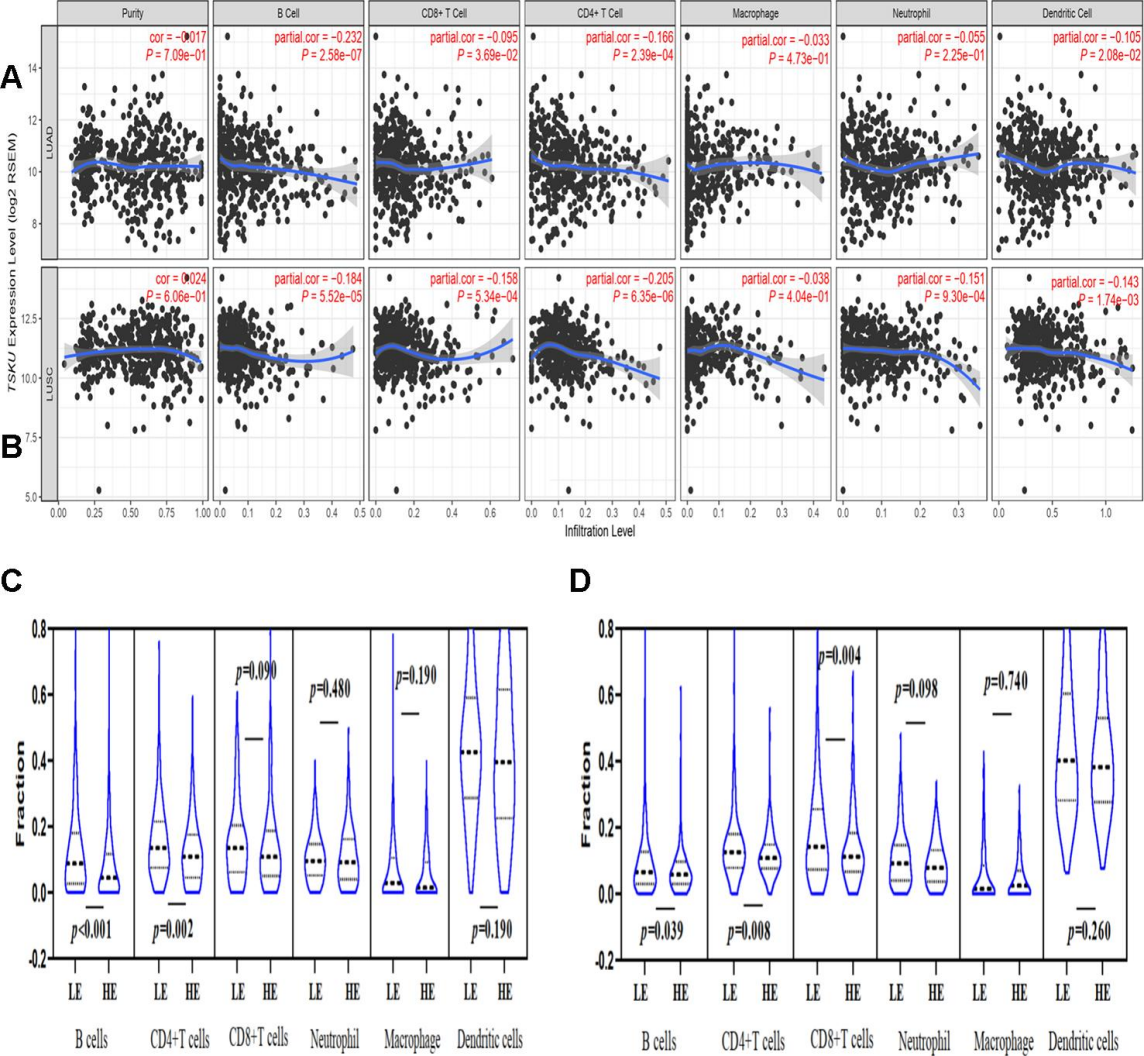
**Correlations of *TSKU* expression with immune infiltration levels in LUAD and LUSC using TIMER database.** (**A**) The correlations of *TSKU* expression with infiltrating levels of B cells, CD8^+^ T cells, CD4^+^ T cells, and dendritic cells in LUAD (N = 515). (**B**) The correlations of *TSKU* expression with infiltrating levels of B cells, CD8^+^ T cells, CD4^+^ T cells, neutrophils, and dendritic cells in LUSC (N = 501). (**C**) Comparing the proportions of different TIICs between groups with high *TSKU* expression levels (N = 238) and low *TSKU* expression levels (N = 238) in LUAD samples. (**D**) Comparing the proportions of different TIICs between groups with high *TSKU* expression levels (N = 241) and low *TSKU* expression levels (N = 241) in LUSC samples (LE, low expression; HE, high expression).

We further analyzed the correlation of *TSKU* expression with the immune infiltration levels of different cells in NSCLC, including LUAD and LUSC, and found that the expression level of *TSKU* significantly correlated with the levels of infiltrating B cells (cor=-0.232, *P*=2.58e-07), CD4^+^ T cells (cor =-0.166, *P*=2.39e-04), dendritic cells (cor =-0.105, *P*=2.08e-02), and CD8^+^ T cells (cor =-0.095, *P*=3.69e-02) in LUAD (Figure 3A). Meanwhile, the *TSKU* expression level also correlated with the levels of infiltrating B cells (cor =-0.184, *P*=5.52e-05), CD4^+^ T cells (cor =-0.205, *P*=6.35e-06), neutrophil (cor =-0.151, *P*=9.30e-04), DCs (dendritic cells) (cor =-0.143, *P*=1.74e-03), and CD8^+^ T cells (cor =-0.158, *P*=5.34e-04) in LUSC (Figure 3B).

Moreover, we analyzed the proportion of different TIICs between groups with higher and lower *TSKU* expression levels in NSCLC using the TIMER database. The samples with high *TSKU* expression had a lower infiltration level of B cells and CD4^+^ T cells than the samples with low *TSKU* expression in LUAD and LUSC (Figure 3C, 3D).

### Correlation between *TSKU* expression and gene markers of TIICs in lung cancer

**Figure 4:**
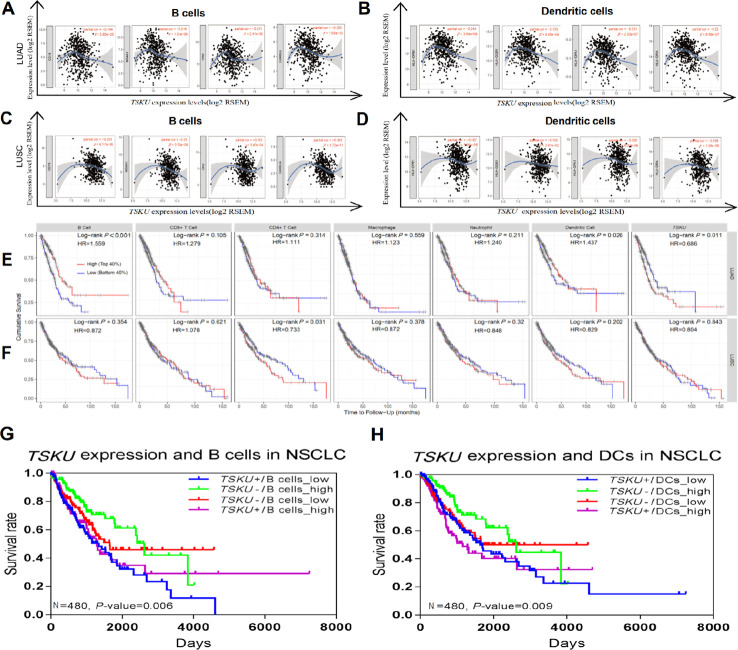
**Correlations between *TSKU* expression and infiltrating B cell and DC levels in LUAD and LUSC.** Gene markers include CD19, CD20, CD21, and CD40L for B cells; HLA-DPB1, HLA-DQB1, and HLA-DRA for dendritic cells. (**A**, **B**) Scatterplots of correlations between *TSKU* expression and gene markers of B cells (**A**) and DCs (**B**) in LUAD (N = 515). (**C**, **D**) Scatterplots of correlations between *TSKU* expression and gene markers of B cells (**C**) and DCs (**D**) in LUSC (N = 501). (**E**) Patients with low infiltrating levels of B cell and dendritic cell showed a poor survival in LUAD (B cell, N =496) (dendritic cell, N =501). (**F**) Patients with high infiltrating levels of CD4^+^ T cell showed a poor survival in LUSC (CD4^+^ T cell, N =492). (**G**) The survival of patients with high or low *TSKU* expression and high or low infiltrating B cell levels in NSCLC (N=480). (**H**) The survival of patients with high or low *TSKU* expression and high or low infiltrating DC levels in NSCLC (N=480). (The marked blue means high *TSKU* expression and low B cells (or DCs) infiltration (N=120); marked green means low *TSKU* expression and high B cells (or DCs) infiltration (N=120); marked red means low *TSKU* expression and low B cells (or DCs) infiltration (N=120); marked purple means high *TSKU* expression and high B cells (or DCs) infiltration (N=120)).

Interestingly, while analyzing the relationships between *TSKU* expression and the marker genes of different immune cells, including CD8^+^ T cells, T cells (general), B cells, monocytes, TAMs, M1 and M2 macrophages, neutrophils, NK (natural killer) cells, DCs, exhausted T cells, and different subtypes of CD4^+^ T cells (T helper 1 (Th1) cells, T helper 2 (Th2) cells, follicular helper T (Tfh) cells, Th17 cells, and Tregs) in LUAD and LUSC (Table 1), we found that most of the gene markers of B cells and DCs significantly correlated with *TSKU* expression levels, especially CD19, CD20, CD21, and CD40L for B cells and HLA-DPB1, HLA-DQB1, HLA-DRA, and HLA-DPA1 for DCs (Figure 4A–4D).

**Table 1:** Correlations between *TSKU* expression and markers of TIICs in the TIMER database.

**Immune cells**	**Gene markers**	**LUAD**				**LUSC**			
		**None**		**Purity**		**None**		**Purity**	
		**Cor**	***P***	**Cor**	***P***	**Cor**	***P***	**Cor**	***P***
CD8^+^ T cell	CD8A	-0.034	0.440	-0.057	0.210	-0.230	***	-0.230	***
	CD8B	-0.035	0.430	-0.050	0.270	-0.288	***	-0.283	***
T cell	CD3D	-0.081	0.068	-0.107	*	-0.241	***	-0.250	***
(general)	CD3E	-0.111	*	-0.148	**	-0.220	***	-0.234	***
	CD2	-0.116	*	-0.145	*	-0.232	***	-0.240	***
B cell	CD19	-0.138	**	-0.184	***	-0.182	***	-0.203	***
	CD20 (MS4A1)	-0.177	***	-0.216	***	-0.223	***	-0.250	***
	CD21 (CR2)	-0.193	***	-0.211	***	-0.154	**	-0.163	**
	CD40L (CD40LG)	-0.258	***	-0.283	***	-0.271	***	-0.302	***
Monocyte	CD86	-0.081	0.066	-0.094	*	-0.171	**	-0.167	**
	CD115 (CSF1R)	-0.084	0.058	-0.084	0.064	-0.079	0.078	-0.060	0.190
TAM	CCL2	-0.064	0.130	-0.064	0.160	-0.151	**	-0.138	*
	CD68	0.060	0.170	0.064	0.150	0.052	0.240	0.079	0.083
	IL10	-0.058	0.190	-0.060	0.180	-0.132	*	-0.120	*
M1	INOS (NOS2)	0.034	0.440	0.014	0.750	-0.180	***	-0.190	***
Macrophage	IRF5	0.032	0.470	0.039	0.390	0.116	*	0.139	*
	COX2 (PTGS2)	-0.091	*	-0.103	*	-0.121	*	-0.123	*
M2	CD163	-0.029	0.510	-0.034	0.450	-0.088	0.050	-0.075	0.100
Macrophage	VSIG4	-0.060	0.170	-0.061	0.180	-0.074	0.098	-0.056	0.220
	MS4A4A	-0.097	*	-0.103	*	-0.105	*	-0.089	0.050
Neutrophils	CD66b (CEACAM8)	-0.263	***	-0.253	***	-0.076	0.090	-0.072	0.120
	CD11b (ITGAM)	-0.128	*	-0.125	*	-0.152	**	-0.152	**
	CCR7	-0.173	***	-0.204	***	-0.255	***	-0.274	***
Natural	KIR2DL1	0.013	0.780	0.007	0.870	-0.074	0.090	-0.072	0.120
killer	KIR2DL3	0.067	0.130	0.059	0.190	-0.104	*	-0.100	*
	KIR2DL4	0.132	*	0.114	*	0.036	0.430	0.053	0.250
	KIR3DL1	0.009	0.850	0.007	0.870	-0.096	*	-0.081	0.078
Dendritic	HLA-DPB1	-0.223	***	-0.244	***	-0.179	***	-0.187	***
cell	HLA-DQB1	-0.146	**	-0.159	**	-0.108	*	-0.102	*
	HLA-DRA	-0.203	***	-0.220	***	-0.197	***	-0.198	***
	HLA-DPA1	-0.218	***	-0.231	***	-0.199	***	-0.205	***
	BDCA-1 (CD1C)	-0.311	***	-0.305	***	-0.065	0.150	-0.052	0.260
	BDCA-4 (NRP1)	-0.178	***	-0.175	***	0.077	0.084	0.107	*
Th1	T-bet (TBX21)	-0.042	0.340	-0.062	0.170	-0.235	***	-0.245	***
	STAT4	-0.113	*	-0.129	*	-0.209	***	-0.219	***
	STAT1	0.131	*	0.128	*	-0.063	0.160	-0.057	0.210
	IFN-γ (IFNG)	0.084	0.056	0.076	0.090	-0.166	**	-0.159	**
	TNF-α (TNF)	-0.103	*	-0.101	*	-0.159	**	-0.159	**
Th2	GATA3	0.035	0.430	0.047	0.300	-0.143	*	-0.150	**
	STAT6	-0.122	*	-0.124	*	-0.084	0.062	-0.095	*
	STAT5A	-0.093	*	-0.097	*	-0.185	***	-0.186	***
	IL13	-0.088	*	-0.087	0.054	-0.159	**	-0.150	**
Tfh	BCL6	-0.176	***	-0.171	**	0.106	*	0.099	*
	IL21	-0.033	0.450	-0.040	0.370	-0.148	**	-0.134	*
Th17	STAT3	-0.075	0.095	-0.075	0.098	0.007	0.880	0.007	0.880
	IL17A	-0.036	0.420	-0.054	0.230	-0.115	*	-0.115	*
Treg	FOXP3	-0.024	0.590	-0.043	0.360	-0.178	***	-0.181	***
	CCR8	-0.116	*	-0.130	*	-0.177	***	-0.170	**
	STAT5B	-0.186	***	-0.188	***	-0.043	0.330	-0.030	0.510
	TGFβ (TGFB1)	-0.177	***	-0.186	***	0.154	**	0.162	**
T cell	PD-1 (PDCD1)	0.035	0.420	0.021	0.640	-0.206	***	-0.212	***
exhaustion	CTLA4	-0.097	*	-0.122	*	-0.211	***	-0.219	***
	LAG3	0.083	0.059	0.074	0.100	-0.175	***	-0.172	**
	TIM-3 (HAVCR2)	-0.035	0.430	-0.040	0.370	-0.125	*	-0.110	*
	GZMB	0.141	*	0.132	*	-0.180	***	-0.182	***

LUAD, lung adenocarcinoma; LUSC, lung squamous cell carcinoma; TAM, tumor-associated macrophage; Th, T helper cell; Tfh, Follicular helper T cell; Treg, regulatory T cell; Cor(r), value of Spearman’s correlation; None, correlation without adjustment. Purity, correlation adjusted by tumor purity. *P < 0.05; **P < 0.001; ***P < 0.0001.

### Prognostication of different NSCLC subtypes defined by the combination of *TSKU* expression and infiltrating B cell (or DC) levels

Tumor-infiltrating lymphocytes, which are identified as an independent predictor of survival, have the potential to affect cancer prognosis [22, 23]. Therefore, we analyzed the impact of TIICs on the prognosis of NSCLC patients and found that patients with low levels of infiltrating B cell (HR=1.559; 95% CI, 1.179-2.062, Cox *P*<0.001) and DC (HR=1.437; 95% CI, 1.041-1.984, Cox *P*=0.026) presented a poorer prognosis in LUAD than patients with high levels of infiltrating B cell and DC (Figure 4E). However, the infiltration level of B cells (HR=0.872; 95% CI, 0.645-1.180, Cox *P*=0.354) and DCs (HR=0.829; 95% CI, 0.618-1.113, Cox *P*=0.202) have no associated significantly with the prognosis in LUSC (Figure 4F). Based on the association of infiltrating B cell and DC levels with prognosis in LUAD, we further explored whether the combined analysis of *TSKU* expression and infiltrating B cell (or DC) levels yielded different prognoses in NSCLC patients. Patients with high *TSKU* expression and low infiltrating B cell levels had poorer survival than those with low *TSKU* expression and high infiltrating B cell levels (HR=2.016; 95% CI, 1.330-3.057, Cox *P*=0.001) (Figure 4G). A similar result was observed with infiltrating DC levels (HR=1.678; 95% CI, 1.080-2.607, Cox *P*=0.021) (Figure 4H). Regardless of the disease subtype (LUAD or LUSC), patients with high TSKU expression and low infiltrating B cell levels presented a poorer survival than those with low TSKU expression and high infiltrating B cell levels. However, high or low *TSKU* expression and infiltrating DC levels did not affect the prognosis of patients in either LUAD or LUSC datasets (Supplementary Figure 3). These data suggest that the combination of high *TSKU* expression and low infiltrating B cell levels may be associated with a poor prognosis in NSCLC patients.

### Correlation between *TSKU* promoter hypomethylation and elevated *TSKU* expression in NSCLC

**Figure 5:**
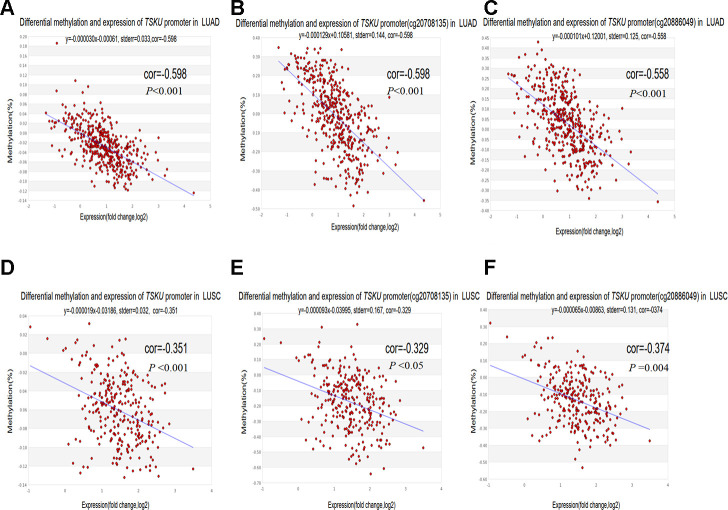
**Correlations between differential *TSKU* methylation and expression in LUAD and LUSC.** TCGA Infinium 450K methylation probes in the promoter region, including the cg20708175 and cg20886049 probes; (**A**–**C**) Scatterplots of correlations between differential *TSKU* methylation and expression level of all CpG sites (probes) in the promoter (**A**), cg20708175 (**B**), and cg20886049 (**C**) in LUAD (N = 471). (**D**–**F**) Scatterplots of correlations between differential *TSKU* methylation and expression level of all CpG sites (probes) in the promoter (**D**), cg20708175 (**E**), and cg20886049 (**F**) in LUSC (N = 406). Cor(r): the value determined by calculating the Pearson correlation coefficient.

To clarify whether the aberrant methylation of the promoter affects gene expression, we evaluated the correlation between the *TSKU* methylation level in the promoter region and its expression. There were quite a few probes in the promoter regions with a negative correlation between methylation and expression for *TSKU* in LUAD and LUSC, as analyzed by MEXPRESS (Supplementary Figure 4). We further analyzed the correlation of *TSKU* methylation with the expression level in LUAD and LUSC datasets from TCGA data using the MethHC database. There were significant negative correlations between differential *TSKU* methylation and expression level of all CpG sites (probes) in the promoter in LUAD (cor =-0.598, *P* <0.001) and LUSC (cor =-0.351, *P* <0.001) datasets (Figure 5A, 5D). There were significant negative correlations between differential methylation and expression for some probes in the promoter region in LUAD, including cg20708135 (cor =-0.598, *P* <0.001) and cg20886049 (cor =-0.558, *P* <0.001) (Figure 5B, 5C). In addition, a similar trend was observed in LUSC including the cg20708135 (cor =-0.329, *P* <0.05) and cg20886049 (cor =-0.374 *P* =0.004) probes (Figure 5E, 5F).

### Correlation between *TSKU* methylation and the proportion of infiltrating immune cells in LUAD and LUSC

**Figure 6:**
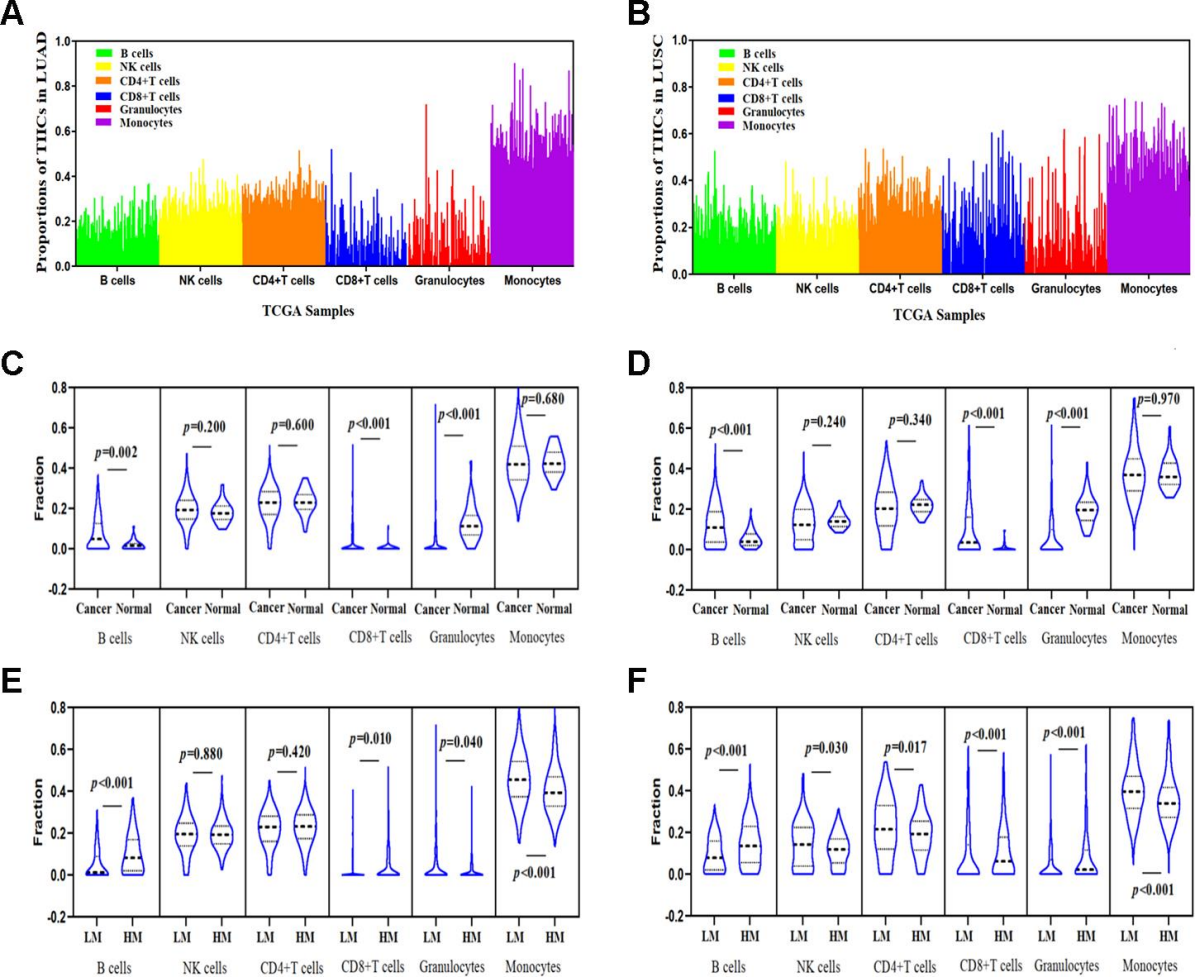
**Correlations between *TSKU* methylation and the proportions of infiltrating immune cells in LUAD and LUSC.** (**A**) The proportions of tumor-infiltrating immune cells (TIICs) in every sample using the TCGA Infinium 450K methylation data in LUAD (N = 460). (**B**) The proportions of TIICs in every sample using the TCGA Infinium 450K methylation data in LUSC (N = 372). (**C**) Comparing the proportions of TIICs in tumor tissues (N = 460) and normal tissues (N = 32) in LUAD datasets. (**D**) Comparing the proportions of TIICs in tumor tissues (N = 372) and normal tissue (N = 43) in LUSC datasets. (**E**) Comparing the proportions of different TIICs between groups with high *TSKU* methylation levels (N = 230) and low *TSKU* methylation levels (N = 230) in LUAD samples. (**F**) Comparing the proportions of different TIICs between groups with high *TSKU* methylation levels (N = 185) and low *TSKU* methylation levels (N = 185) in LUSC samples (LM, low methylation; HM, high methylation).

We calculated the proportion of infiltrating immune cells in every sample using the EpiDISH (Epigenetic Dissection of Intra Sample Heterogeneity) algorithm and TCGA Infinium 450K methylation data in LUAD and LUSC (Figure 6A, 6B) datasets and found that cancer tissues contained a higher proportion of infiltrating B cells, NKs, CD4^+^ T cells, and granulocytes than of CD8^+^ T cells and monocytes in both LUAD and LUSC datasets. Furthermore, the abundance of B cells and CD8^+^ T cells in cancer tissues were significantly higher than those in normal tissues. Nevertheless, the level of granulocytes in cancer tissues was lower than that in normal tissues (Figure 6C, 6D).

We further evaluated the proportion of different TIICs between groups with higher and lower *TSKU* methylation levels in LUAD and LUSC samples from TCGA datasets (Figure 6E, 6F) and found that the proportion of B cells in cancer tissues with *TSKU* hypermethylation was higher than that in cancer tissues with *TSKU* hypomethylation. However, the level of monocytes was higher in hypomethylated samples than that in hypermethylated samples.

### *TSKU* methylation status and prognosis in different cancers

In light of the significant negative correlation between differential methylation and expression, we further analyzed the association between methylation level of *TSKU* and overall survival in 24 types of cancer from TCGA data via the MethSurv database (Supplementary Table 3). We found that low *TSKU* methylation was associated with poor prognosis in ACC, BRCA, KICH, LGG (brain lower grade glioma), and PAAD, while high *TSKU* methylation was associated with good prognosis in KIRC and UCEC (uterine corpus endometrial carcinoma). However, there was no significant association between *TSKU* methylation and prognosis in LUAD (HR=0.824, Cox *P*=0.240; HR=0.866, Cox *P*=0.420) and LUSC (HR=1.198, Cox *P*=0.360; HR=1.338, Cox *P*=0.150).

## DISCUSSION

In this study, we found for the first time that the levels of *TSKU* methylation and expression significantly correlated with tumor-infiltrating B cell levels in NSCLC. In addition, high *TSKU* expression combined with low tumor-infiltrating B cell levels may influence the prognosis of patients with NSCLC.

According to the Oncomine and TIMER databases, we found consistent results on the differential *TSKU* expression between tumor and normal tissues for the lung, breast, kidney, and liver cancer (Figures 1A, 1B). We further analyzed the association between *TSKU* expression and the prognosis of these cancers and found that, only in lung cancer, the high expression of *TSKU* was associated with a poor OS based on the above results of *TSKU* expression differential analysis (Figure 2A, 2B; Supplementary Figure 2A–2G). In addition, we found only research on the functional mechanism of *TSKU* expression in lung cancer [17]. This study will support us to explore the association between *TSKU* expression and the prognosis of lung cancer patients based on public databases. These results suggest that *TSKU* may be a potential independent prognostic biomarker in lung cancer.

In previous studies, TSKU serves as a modulator involved in the wound healing process via inhibition of TGF-β secretion from macrophages (18). Since TGF-β is a pleiotropic cytokine with immunoregulatory properties that activates the differentiation and proliferation of immune cells, *TSKU* may involve in the immunoregulation and relate to the immune infiltrating cells. Therefore, we analyzed the relationship between *TSKU* and the level of tumor immune infiltrating cells to explore whether it is associated with the prognosis of lung cancer. And found that high *TSKU* expression correlated with low B cell and CD4^+^ T cell infiltration levels in both LUAD and LUSC (Figure 3A–3D). Moreover, we also observed correlations between *TSKU* expression and gene markers of B cells and DCs, which demonstrated that *TSKU* expression might play a role in regulating tumor immunity in both LUAD and LUSC (Table 1). Although these correlations between *TSKU* expression and gene markers were not very strong, the low levels of B cell and DC infiltration, and mainly of B cell infiltration, were associated with poor prognosis in LUAD (Figure 4E). We further found that the combination of high *TSKU* expression and low B cell infiltration identified a group of patients with poor survival in NSCLC (Figure 4G). These results suggest that the co-assessment of *TSKU* expression and B cell infiltration levels may provide a useful assessment of the immunologic state in NSCLC and, in turn, the patient survival.

Recent studies have focused on the possible mechanisms that may explain why elevated *TSKU* expression and a low level of infiltrating B cells are associated with poor survival in NSCLC. TSKU, a 37 kDa core protein, is a prototype class IV SLRP that is considered a structural element of the extracellular matrix (ECM) [24]. Similar to TSKU, decorin (DCN) and biglycan (BGN) are two key SLRPs that have altered expression in various cancers with diverse clinical outcomes, and *BGN* serves as a potential marker of cancer proliferation associated with poor clinical outcome [25–27]. Moreover, the expression of CD40, serving as a marker of DLBC, is co-expressed with *BGN* and associated with a superior prognosis [28]. The previous study also confirmed that *TSKU* is more highly expressed in their lung cancer tissue (N=62) and cells and activates proliferation in cancer cells [17]. Therefore, *TSKU* expression may be related to clinical outcome development and may be indicative of a potential mechanism in which *TSKU* regulates B cell functions in NSCLC. Nevertheless, the mechanisms behind high *TSKU* expression leading to poorer survival in NSCLC patients with low levels of infiltrating B cell need to be studied further.

Another important aspect of this study was the significant negative correlation between differential methylation and expression in the promoter region (probes cg20708135 and cg20886049) of *TSKU* (Figures 5A–5F). However, we did not observe a significant association between *TSKU* methylation and prognosis in NSCLC (Supplementary Table 3). A possible reason is that methylation does not serve as an independent factor regulating gene expression. Other factors, including copy number alterations, transcription factor production and recruitment, histone modifications, and microRNA expression, may also play a role in regulating *TSKU* expression [29]. In addition, the *TSKU* methylation probes from the TCGA Illumina Infinium HumanMethylation450 BeadChip are limited and do not include all probes to analyze the effects on prognosis. Therefore, it is necessary to explore further other factors affecting *TSKU* expression in addition to methylation. Currently, our results preliminarily demonstrate that *TSKU* hypomethylation in the promoter region increases the expression levels of *TSKU* and worsens the clinical outcome of patients. More importantly, we first utilized methylation levels in patients with NSCLC to evaluate the abundance of six types of TIICs (Figure 6A, 6B). The proportion of B cells and CD8^+^ T cells were higher in tumors than in normal tissue (Figure 6C, 6D). According to *TSKU* methylation levels, we further analyzed *TSKU* hypomethylation levels in cancer tissue and found a low proportion of B cells in lung cancer patients (Figure 6E, 6F). These results are consistent with those found during the evaluation of infiltrating B cell levels in samples with high *TSKU* expression.

TILs were identified as a favorable prognostic marker that plays a critical role in shaping tumor development and determining treatment responses in the tumor microenvironment [30]. The reasons for selecting DNA methylation to estimate the composition and purity of TIICs were based on the following studies. First, a previous study demonstrated that DNA methylation might represent a specific biomarker for distinguishing immune cell subtypes [11]. Additionally, in 2019, Loo Yau, H et al. found that the aberrant epigenomes, including methylation alterations, observed in cancer cells and infiltrating immune cells that play a critical role in driving or mediating tumor progression and provide a vulnerability that may be utilized in epigenetic therapy [31]. Recent studies have often utilized DNA methylation data profiled by TCGA to accurately estimate tumor purity and cellular composition, such as MethylCIBERTSORT, EpiDISH, and CP (constrained projection) algorithms. In addition, EpiDISH has robust correlations, and it outperformed both CP and MethylCIBERSORT in terms of estimating mixed cell proportion [32–34]. Therefore, we selected the deconvolution method of EpiDISH to evaluate the intrasample heterogeneity for six types of TIICs. Advances in the deconvolution method to estimate both tumor purity and composition from DNA methylation data might provide some insights that reveal potential biomarkers for immunotherapy response and increase our understanding of the contribution of the tumor microenvironment in lung cancer.

In this study, we first evaluated the abundance of six TIICs in LUAD and LUSC methylation data using the EpiDISH algorithm. More extensive studies to determine the generality and feasibility of the EpiDISH method in other tumor tissues are needed. Additionally, we should further validate whether *TSKU* methylation in the promoter affects the expression of *TSKU* and clinical outcome using large NSCLC patient sample sets.

In summary, *TSKU* overexpression that combines with low infiltrating B cell levels to influence the prognosis of NSCLC patients. Our study provides insights into the potential role of *TSKU* in tumor immunology and its identification as a prognostic biomarker.

## MATERIALS AND METHODS

### Oncomine database analysis

We compared the *TSKU* mRNA levels of multiple cancers with the levels of corresponding normal tissues using the Oncomine database (http://www.oncomine.org). The threshold was selected as a *P* value=1E-5, with a 1.5-fold change.

### Prognoscan database analysis

The associations between the expression of *TSKU* and survival in various types of cancer were analyzed using the PrognoScan database (http://www.abren.net/PrognoScan/) [35]. The significance threshold was a Cox *P*-value< 0.05.

### TIMER database analysis

TIMER is an integrative database that analyzes immune infiltrates in different cancer types (https://cistrome.shinyapps.io/timer), including information on TIICs in over 10,000 tumor samples across 32 cancer types from TCGA data, by applying a statistical deconvolution method to estimate the abundance of TIICs from gene expression profiles [36, 37]. We first validated the differential *TSKU* expression between tumor and normal tissues using the Oncomine database analysis. Then, we further analyzed the correlations between expression of *TSKU* and the abundance of infiltrating immune cells, including B cells, CD4^+^ T cells, CD8^+^ T cells, neutrophils, macrophages, and dendritic cells, in different cancer tissues and analyzed the association of TIICs with the prognosis of lung cancer patients. The correlation between *TSKU* expression and gene markers of TIICs (CD8^+^ T cells, T cells (general), B cells, monocytes, TAMs, M1 macrophages, M2 macrophages, neutrophils, NK cells, DCs, Th1 cells, Th2 cells, Tfh cells, Th17 cells, Tregs, and exhausted T cells) were estimated by Spearman's correlation [38, 39].

### GEPIA database analysis

We validated the associations between *TSKU* expression levels and prognosis in multiple cancers using the GEPIA database (http://gepia.cancer-pku.cn/index.html) [40].

### MethHC database analysis

The MethHC database (http://awi.cuhk.edu.cn/~MethHC/methhc_2020/php/index.php) integrates data regarding DNA methylation, gene expression, and the correlations between methylation and gene expression for different cancers of TCGA [41]. We analyzed the correlation between differential methylation and expression of *TSKU* in both LUAD and LUSC datasets using the MethHC database.

### MEXPRESS database analysis

MEXPRESS is a data visualization tool designed for the easy visualization of TCGA expression, DNA methylation, and clinical data (http://mexpress.be/) [42]. We analyzed the methylation of *TSKU* with probes distributed in different regions and visualized the correlation between *TSKU* methylation and expression via the localization of each probe.

### MethSurv database analysis

The MethSurv database (https://biit.cs.ut.ee/methsurv/) performs univariable and multivariable survival analysis based on DNA methylation data from TCGA [43]. We evaluated the associations between methylation levels of *TSKU* and prognosis in multiple tumor types.

### EpiDISH package analysis

EpiDISH is an R package for inferring the proportions of a priori known cell subtypes present in a sample representing a mixture of such cell types. This package identifies differentially methylated cell types and the direction of their methylation change, including six cell subtypes (B cells, CD4^+^ T cells, CD8^+^ T cells, NK cells, monocytes, and granulocytes; noting that granulocytes consist of neutrophils and eosinophils) [32, 34]. We assessed the proportion of six tumor-infiltrating cells in the tumor and normal tissues of lung cancer patients using the EpiDISH algorithm via the TCGA Infinium Human Methylation 450K arrays. According to the abundance of the six immune cells in every patient, we evaluated the proportions of different TIICs between groups with higher and lower *TSKU* methylation levels in LUAD and LUSC datasets.

### Statistical analysis

The proportion of immune cell tumors estimated by gene expression data was downloaded by the TIMER database and HumanMethylation450 data to quantify immune infiltration analysis were downloaded by the TCGA lung cancer dataset from the NCI GDC data. These results were analyzed using the R statistical package (R version 3.5.2) and GraphPad Prism 8.00 software (La Jolla, CA, USA). All *P* values were two-sided, and *P* values <0.05 were considered statistically significant for all statistical analyses.

## Supplementary Material

Supplementary Figures

Supplementary Tables
