# Supplementary material for: Tsukushi is a novel prognostic biomarker and correlates with tumor-infiltrating B cells in non-small cell lung cancer
Source: Aging (Albany NY). 2021 Jan 10;13(3):4428–51. doi: 10.18632/aging.202403 (PMC7906171; doi:10.18632/aging.202403)
Supplement: Supplementary Figures [file aging-13-202403-s001.pdf]

SUPPLEMENTARY FIGURES

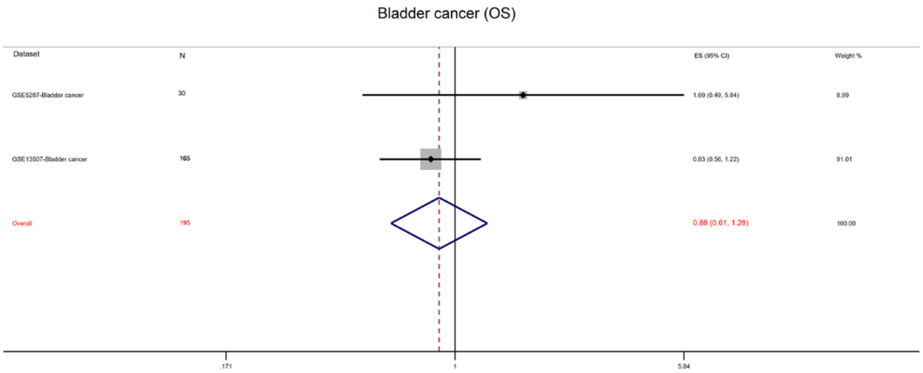

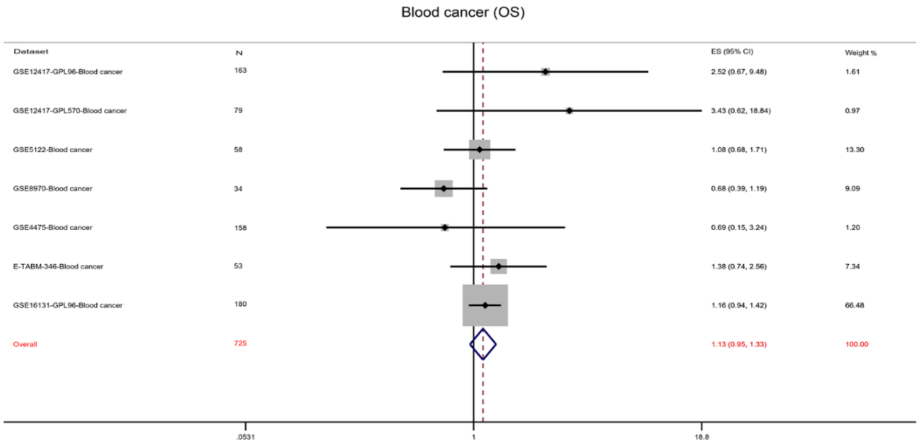

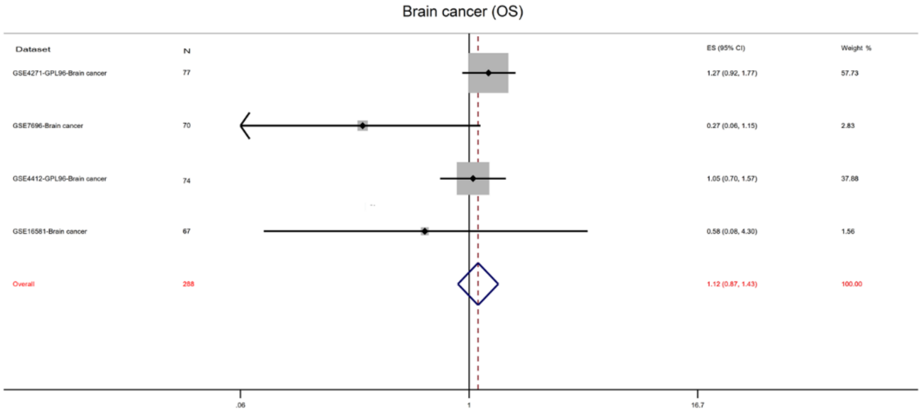

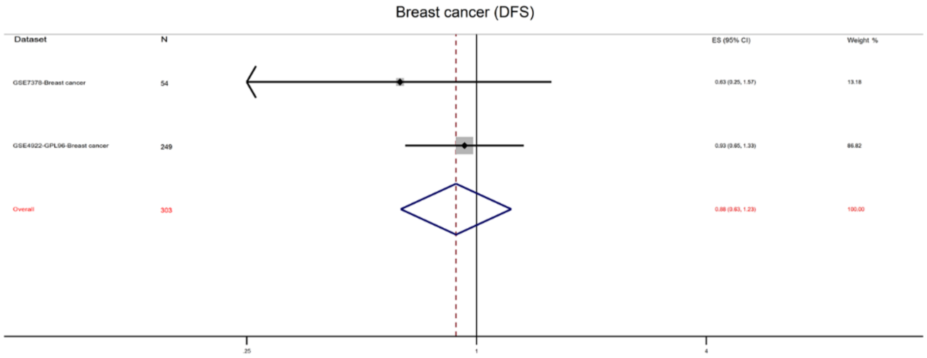

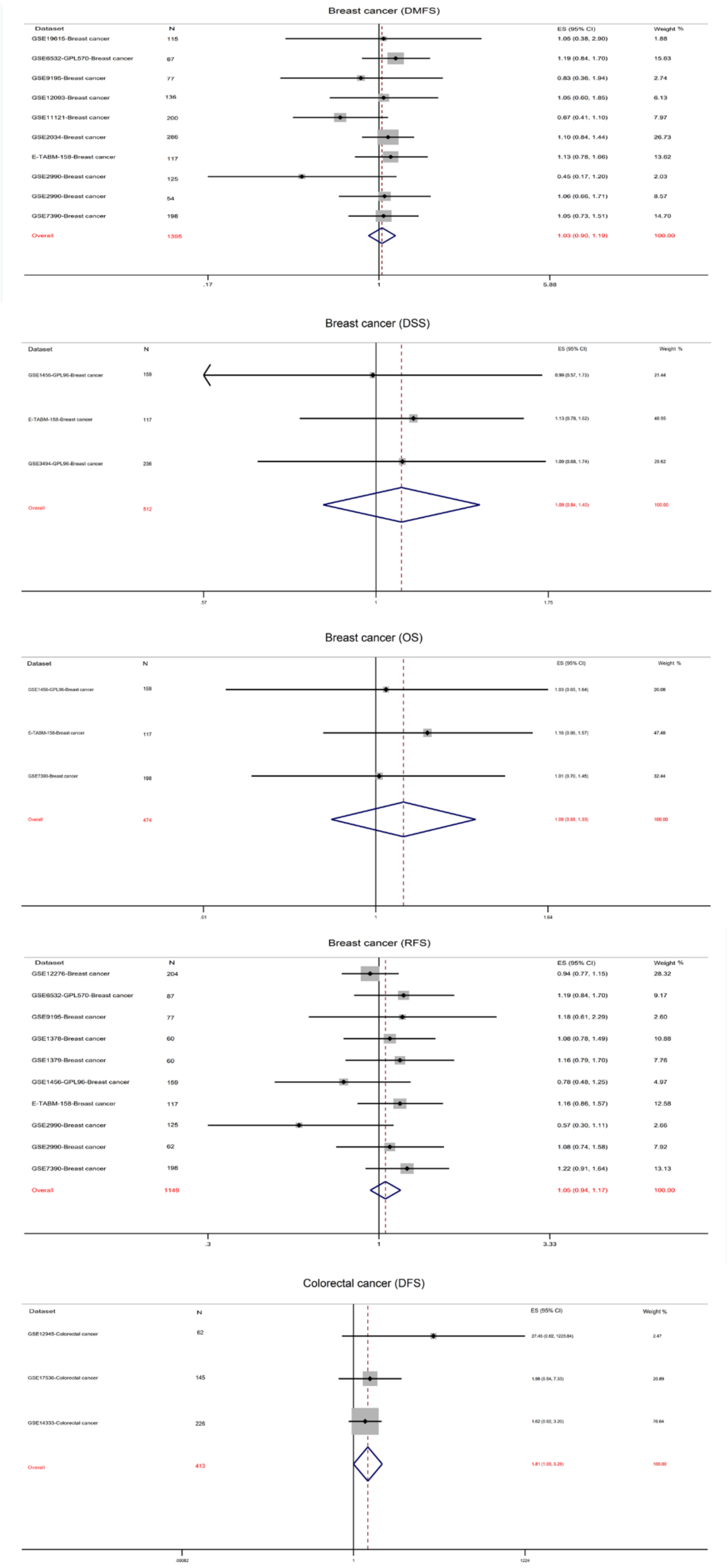
Breast cancer (DMFS)
Dataset
N
ES (95% CI)
Weight %
GSE19615-Breast cancer
GSE6532-GPL570-Breast cancer
GSE9195-Breast cancer
GSE12093-Breast cancer
GSE11121-Breast cancer
GSE2034-Breast cancer
E-TABM-158-Breast cancer
GSE2990-Breast cancer
GSE7390-Breast cancer
Overall
Breast cancer (DSS)
Breast cancer (OS)
Breast cancer (RFS)
GSE12276-Breast cancer
GSE1378-Breast cancer
GSE1379-Breast cancer
GSE1456-GPL96-Breast cancer
Colorectal cancer (DFS)

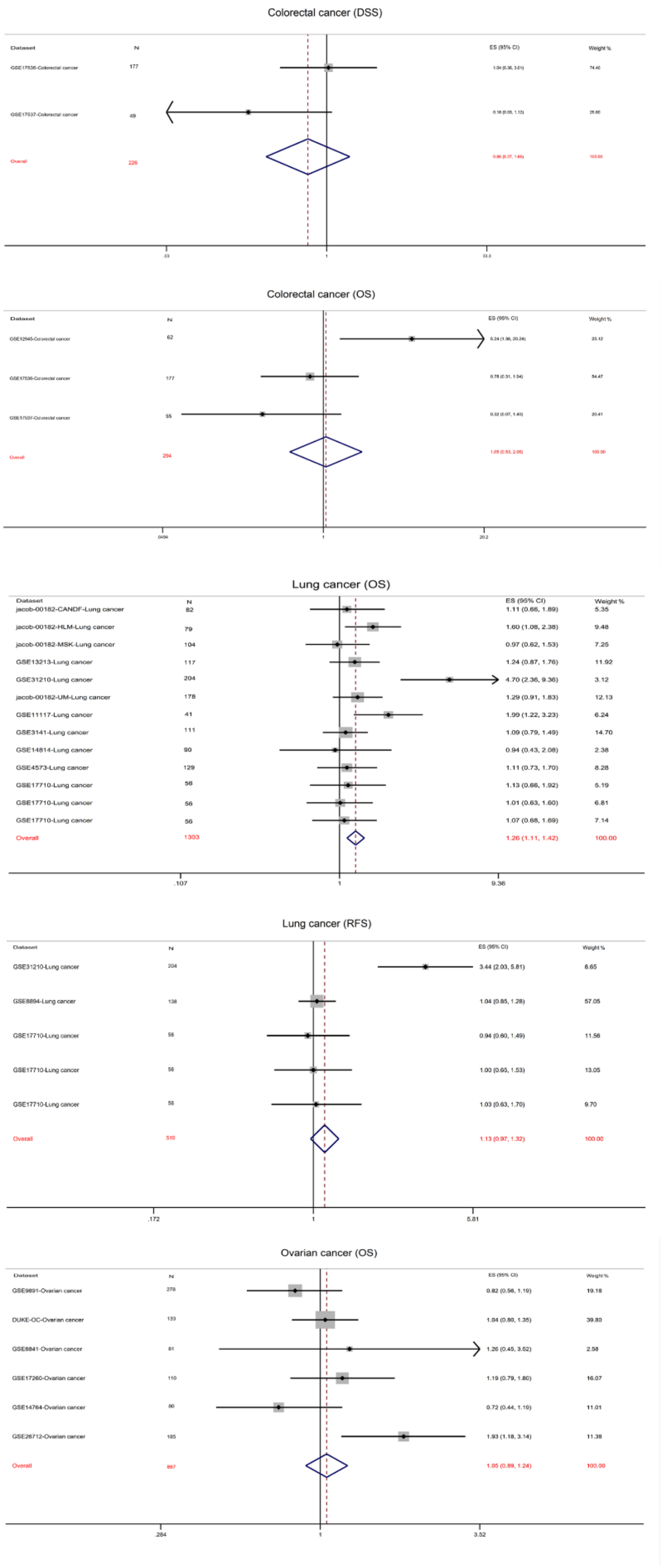

Supplementary Figure 1. Meta-analysis of the associations of TSKU expression with prognosis in different types of cancer from the PrognoScan database. OS, Overall Survival; DSS, Disease Specific Survival; RFS, Relapse-free Survival; DFS, Disease-Free Survival; DMFS, Distant Metastasis Free Survival.

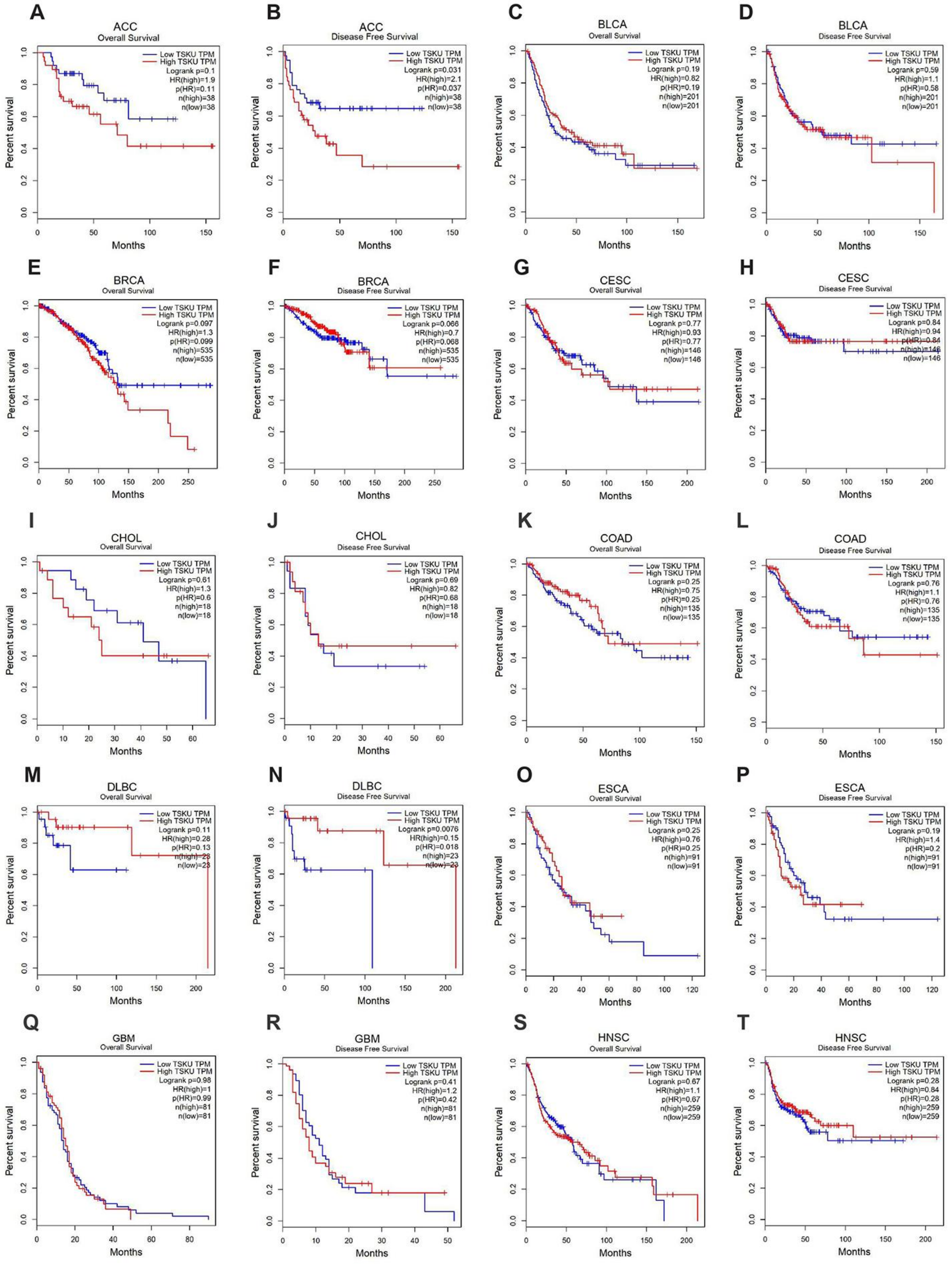

A
ACC
Overall Survival
Low TSKU TPM
High TSKU TPM
Logrank p=0.1
HR(high)=1.9
p(HR)=0.11
n(high)=38
n(low)=38
B
ACC
Disease Free Survival
Low TSKU TPM
High TSKU TPM
Logrank p=0.031
HR(high)=2.1
p(HR)=0.037
n(high)=38
n(low)=38
C
BLCA
Overall Survival
Low TSKU TPM
High TSKU TPM
Logrank p=0.19
HR(high)=0.82
p(HR)=0.19
n(high)=201
n(low)=201
D
BLCA
Disease Free Survival
Low TSKU TPM
High TSKU TPM
Logrank p=0.59
HR(high)=1.1
p(HR)=0.58
n(high)=201
n(low)=201
E
BRCA
Overall Survival
Low TSKU TPM
High TSKU TPM
Logrank p=0.097
HR(high)=1.3
p(HR)=0.099
n(high)=535
n(low)=535
F
BRCA
Disease Free Survival
Low TSKU TPM
High TSKU TPM
Logrank p=0.066
HR(high)=0.7
p(HR)=0.068
n(high)=535
n(low)=535
G
CESC
Overall Survival
Low TSKU TPM
High TSKU TPM
Logrank p=0.77
HR(high)=0.93
p(HR)=0.77
n(high)=146
n(low)=146
H
CESC
Disease Free Survival
Low TSKU TPM
High TSKU TPM
Logrank p=0.84
HR(high)=0.94
p(HR)=0.84
n(high)=146
n(low)=146
I
CHOL
Overall Survival
Low TSKU TPM
High TSKU TPM
Logrank p=0.61
HR(high)=1.3
p(HR)=0.6
n(high)=18
n(low)=18
J
CHOL
Disease Free Survival
Low TSKU TPM
High TSKU TPM
Logrank p=0.69
HR(high)=0.82
p(HR)=0.68
n(high)=18
n(low)=18
K
COAD
Overall Survival
Low TSKU TPM
High TSKU TPM
Logrank p=0.25
HR(high)=0.75
p(HR)=0.25
n(high)=135
n(low)=135
L
COAD
Disease Free Survival
Low TSKU TPM
High TSKU TPM
Logrank p=0.76
HR(high)=1.1
p(HR)=0.76
n(high)=135
n(low)=135
M
DLBC
Overall Survival
Low TSKU TPM
High TSKU TPM
Logrank p=0.11
HR(high)=0.28
p(HR)=0.13
n(high)=23
n(low)=23
N
DLBC
Disease Free Survival
Low TSKU TPM
High TSKU TPM
Logrank p=0.0076
HR(high)=0.15
p(HR)=0.018
n(high)=23
n(low)=23
O
ESCA
Overall Survival
Low TSKU TPM
High TSKU TPM
Logrank p=0.25
HR(high)=0.76
p(HR)=0.25
n(high)=91
n(low)=91
P
ESCA
Disease Free Survival
Low TSKU TPM
High TSKU TPM
Logrank p=0.19
HR(high)=1.4
p(HR)=0.2
n(high)=91
n(low)=91
Q
GBM
Overall Survival
Low TSKU TPM
High TSKU TPM
Logrank p=0.98
HR(high)=1
p(HR)=0.99
n(high)=81
n(low)=81
R
GBM
Disease Free Survival
Low TSKU TPM
High TSKU TPM
Logrank p=0.41
HR(high)=1.2
p(HR)=0.42
n(high)=81
n(low)=81
S
HNSC
Overall Survival
Low TSKU TPM
High TSKU TPM
Logrank p=0.67
HR(high)=1.1
p(HR)=0.67
n(high)=259
n(low)=259
T
HNSC
Disease Free Survival
Low TSKU TPM
High TSKU TPM
Logrank p=0.28
HR(high)=0.84
p(HR)=0.28
n(high)=259
n(low)=259
Percent survival
Months

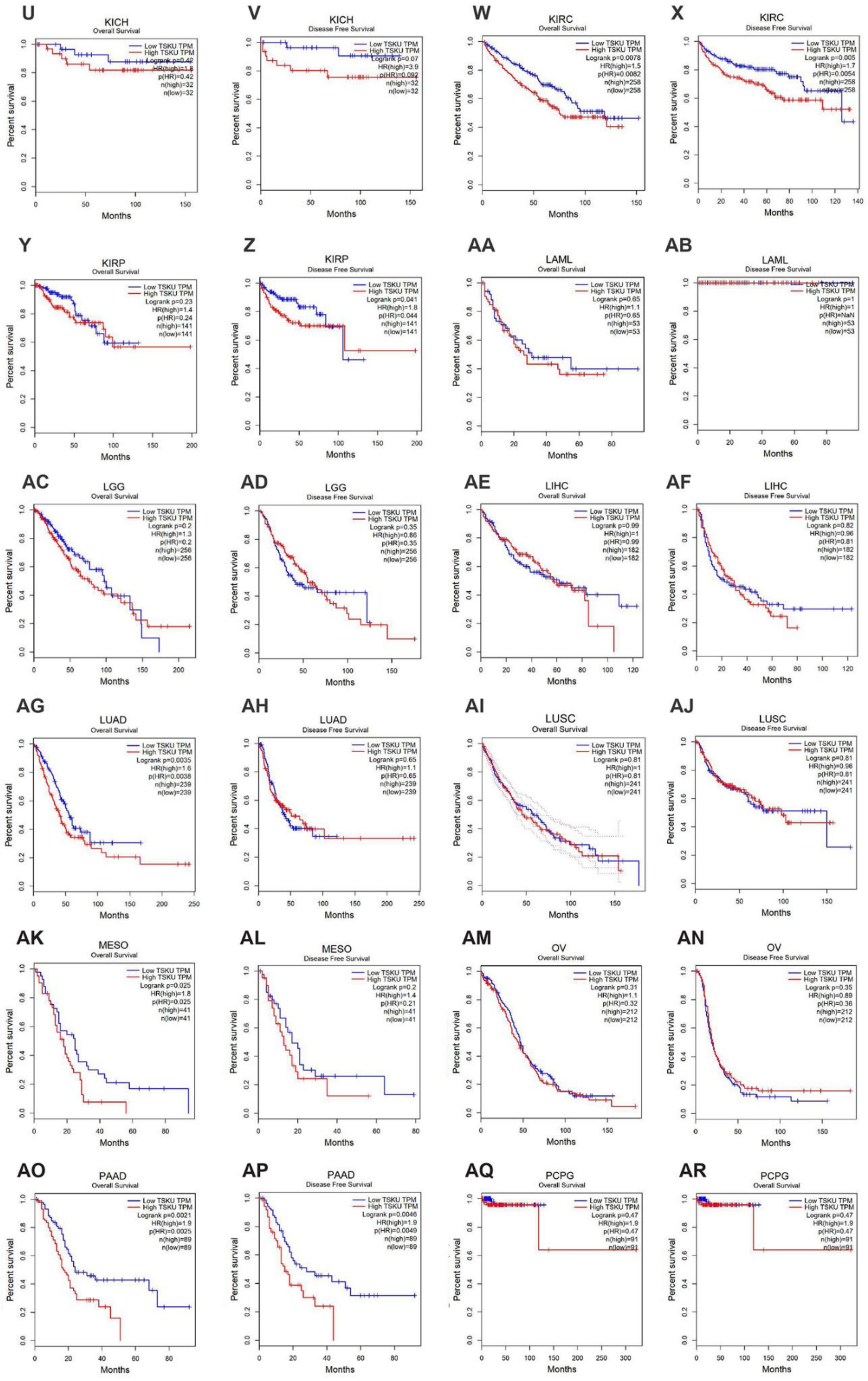

U
KICH
Overall Survival
V
KICH
Disease Free Survival
W
KIRC
Overall Survival
X
KIRC
Disease Free Survival
Y
KIRP
Overall Survival
Z
KIRP
Disease Free Survival
AA
LAML
Overall Survival
AB
LAML
Disease Free Survival
AC
LGG
Overall Survival
AD
LGG
Disease Free Survival
AE
LIHC
Overall Survival
AF
LIHC
Disease Free Survival
AG
LUAD
Overall Survival
AH
LUAD
Disease Free Survival
AI
LUSC
Overall Survival
AJ
LUSC
Disease Free Survival
AK
MESO
Overall Survival
AL
MESO
Disease Free Survival
AM
OV
Overall Survival
AN
OV
Disease Free Survival
AO
PAAD
Overall Survival
AP
PAAD
Disease Free Survival
AQ
PCPG
Overall Survival
AR
PCPG
Overall Survival
Low TSKU TPM
High TSKU TPM
Percent survival
Months

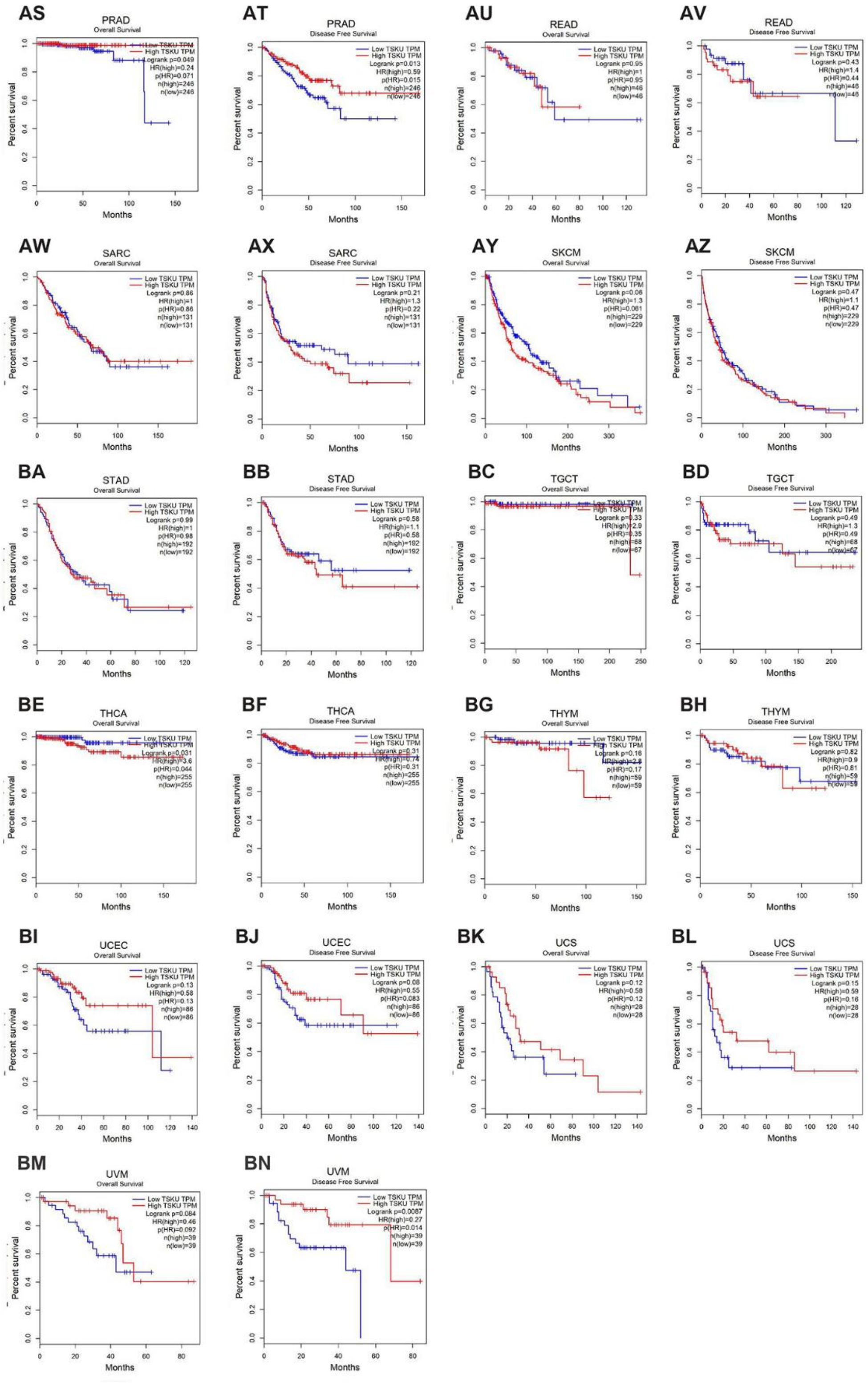

Supplementary Figure 2. Associations of TSKU expression with prognosis in different types of cancer via the GEPIA database. Comparing high and low TSKU expression associated with overall survival curves and disease-free survival curves in adrenocortical carcinoma (ACC) (**A**, **B**) , bladder urothelial carcinoma (BLCA) (**C**, **D**), breast invasive carcinoma (BRCA) (**E**, **F**), cervical squamous cell carcinoma and endocervical adenocarcinoma (CESC) (**G**, **H**), cholangio carcinoma (CHOL) (**I**, **J**), colon adenocarcinoma (COAD) (**K**, **L**), lymphoid neoplasm diffuse large B-cell lymphoma (DLBC) (**M**, **N**), esophageal carcinoma (ESCA) (**O**, **P**), glioblastoma multiforme (GBM) (**Q**, **R**) ,head and neck squamous cell carcinoma (HNSC) (**S**, **T**), kidney chromophobe (KICH) (**U**, **V**), kidney renal clear cell carcinoma (KIRC) (**W**, **X**), kidney renal

papillary cell carcinoma (KIRP) (**Y**, **Z**), acute myeloid leukemia (LAML) (**AA**, **AB**), brain lower grade glioma (LGG) (**AC**, **AD**), liver hepatocellular carcinoma (LIHC) (**AE**, **AF**), lung adenocarcinoma (LUAD) (**AG**, **AH**), lung squamous cell carcinoma (LUSC) (**AI**, **AJ**), mesothelioma (MESO) (**AK**, **AL**), ovarian serous cystadenocarcinoma (OV) (**AM**, **AN**), pancreatic adenocarcinoma (PAAD) (**AO**, **AP**), pheochromocytoma and paraganglioma (PCPG) (**AQ**, **AR**), prostate adenocarcinoma (PRAD) (**AS**, **AT**), rectum adenocarcinoma (READ) (**AU**, **AV**), sarcoma (SARC) (**AW**, **AX**), skin cutaneous melanoma (SKCM) (**AY**, **AZ**), stomach adenocarcinoma (STAD) (**BA**, **BB**), testicular germ cell tumors (TGCT) (**BC**, **BD**), thyroid carcinoma (THCA) (**BE**, **BF**), thymoma (THYM) (**BG**, **BH**), uterine corpus endometrial carcinoma (UCEC) (**BI**, **BJ**), uterine carcinosarcoma (UCS) (**BK**, **BL**), uveal melanoma (UVM) (**BM**, **BN**).

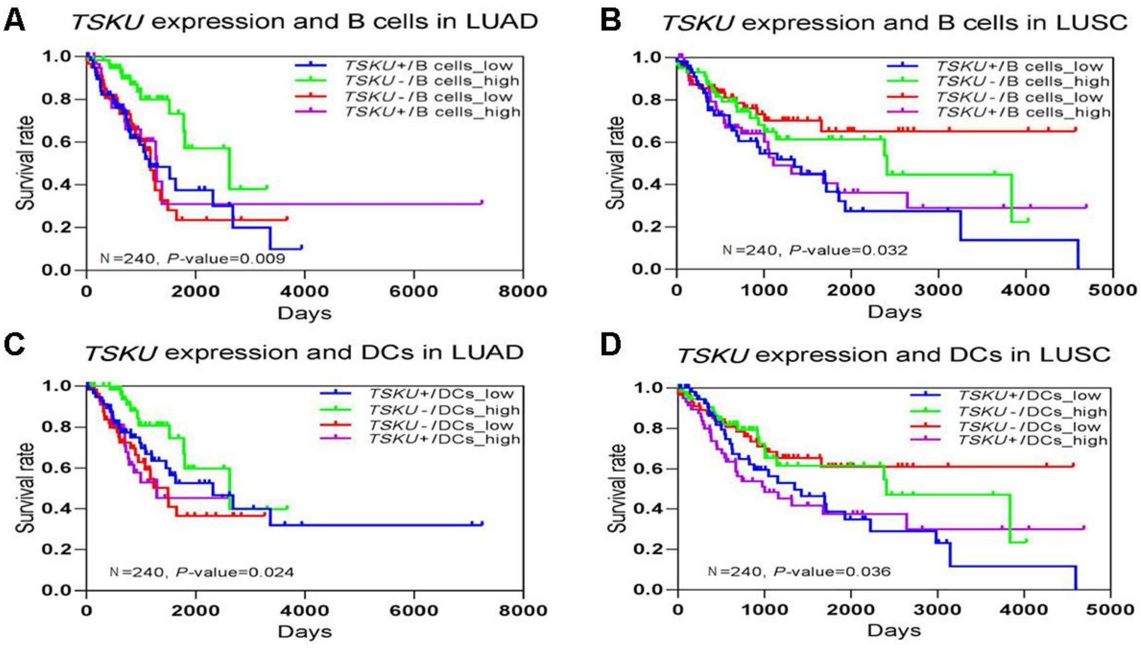

Supplementary Figure 3. The prognosis of different LUAD or LUSC subtypes defined by the combination of TSKU expression and infiltration B cell (or DC) levels. The survival of patients with high or low TSKU expression and high or low infiltrating B cell levels in LUAD (N=240) (**A**) and LUSC (N=240) (**B**). (**C**, **D**) The survival of patients with high or low TSKU expression and high or low infiltrating DC levels in LUAD (N=240) (**C**) and LUSC (N=240) (**D**). (The marked blue means high TSKU expression and low B cells (DCs) infiltration (N=60); marked green means low TSKU expression and high B cells (DCs) infiltration (N=60); marked red means low TSKU expression and low B cells(DCs) infiltration (N=60); marked purple means high TSKU expression and high B cells(DCs) infiltration (N=60).

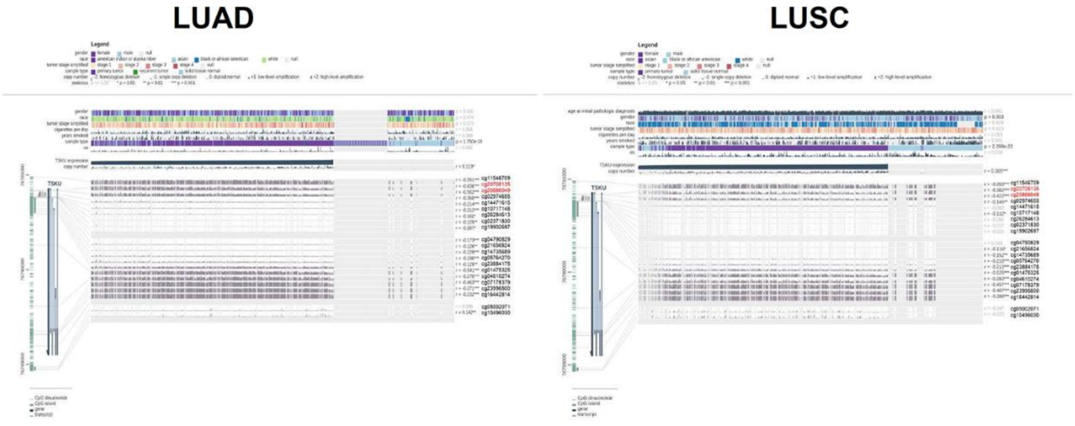

Supplementary Figure 4. Correlations between methylation in all sites (probes) and expression of TSKU in LUAD and LUSC via the MEXPRESS database. TCGA Infinium 450k methylation probes in all sites, including the cg20708135 and cg20886049 probes marked red serving as promoter region; LUAD, lung adenocarcinoma; LUSC, lung squamous cell carcinoma; r value of Pearson correlation; *P < 0.05; **P < 0.001; ***P < 0.0001.
