# Supplementary material for: Tsukushi is a novel prognostic biomarker and correlates with tumor-infiltrating B cells in non-small cell lung cancer
Source: Aging (Albany NY). 2021 Jan 10;13(3):4428–51. doi: 10.18632/aging.202403 (PMC7906171; doi:10.18632/aging.202403)
Supplement: Supplementary Tables [file aging-13-202403-s002.pdf]

SUPPLEMENTARY TABLES

Supplementary Table 1. *TSKU* expression in cancer vs. normal tissue in the oncomine database.

	Cancer types	*P*-value	Fold change	Rank (%)	Sample	Reference (PMID)
Bladder	Superficial Bladder Cancer	7.75E-10	2.406	12%	76	16432078
Brain	Glioblastoma	1.53E-06	7.145	4%	31	16204036
and CNS	Anaplastic Astrocytoma	4.04E-06	2.615	5%	42	16616334
	Oligodendroglioma	2.67E-06	2.661	8%	73	16616334
	Glioblastoma	3.59E-08	3.333	11%	104	16616334
	Brain Glioblastoma	4.85E-07	1.647	14%	552	TCGA
Breast	Tubular Breast Carcinoma	3.30E-10	-1.511	14%	211	22522925
	Medullary Breast Carcinoma	8.57E-06	-1.601	14%	176	22522925
Kidney	Renal Oncocytoma	2.07E-07	-1.542	9%	35	16115910
Liver	Hepatocellular Carcinoma	3.72E-27	-2.136	7%	445	21159642
	Hepatocellular Carcinoma	6.98E-07	-2.328	12%	179	12058060
Lung	Squamous Cell Lung Carcinoma	1.70E-09	1.962	6%	92	20421987
	Lung Adenocarcinoma	4.34E-10	1.952	8%	116	22613842
Sarcoma	Leiomyosarcoma	3.83E-06	-2.424	6%	35	20601955
Others	Yolk Sac Tumor, NOS	8.82E-07	2.723	3%	15	16424014
	Teratoma, NOS	1.65E-08	1.932	3%	20	16424014
	Mixed Germ Cell Tumor, NOS	8.76E-09	1.729	7%	47	16424014

Supplementary Table 2. Associations between TSKU expression and prognosis of different cancers in the prognoScan database.

Cancer type	Dataset	Endpoint	N	Hazard ratio(95%CI)	Cox P
Bladder cancer	GSE5287	OS	30	1.690(0.490 , 5.840)	0.410
	GSE13507	OS	165	0.830(0.560 , 1.220)	0.330
	GSE13507	DSS	165	0.730(0.410 , 1.300)	0.280
Blood cancer	GSE12417-GPL96	OS	163	2.520(0.670 , 9.480)	0.170
	GSE12417-GPL570	OS	79	3.430(0.620 , 18.840)	0.160
	GSE5122	OS	58	1.080(0.680 , 1.710)	0.740
	GSE8970	OS	34	0.680(0.390 , 1.190)	0.180
	GSE4475	OS	158	0.690(0.390 , 1.190)	0.630
	E-TABM-346	OS	53	1.380(0.740 , 2.560)	0.310
	E-TABM-346	EFS	53	1.190(0.660 , 2.150)	0.560
	GSE16131-GPL96	OS	180	1.160(0.940 , 1.420)	0.160
	GSE2658	DSS	559	0.870(0.660 , 1.150)	0.330
Brain cancer	GSE4271-GPL96	OS	77	1.270(0.920 , 1.770)	0.150
	GSE7696	OS	70	0.270(0.060 , 1.150)	0.080
	GSE4412-GPL96	OS	74	1.050(0.700 , 1.570)	0.800
	GSE16581	OS	67	0.590(0.080 , 4.300)	0.590
Breast cancer	GSE19615	DMFS	115	1.050(0.380 , 2.900)	0.920
	GSE12276	RFS	204	0.940(0.770 , 1.150)	0.540
	GSE6532-GPL570	RFS	87	1.190(0.840 , 1.700)	0.330
	GSE6532-GPL570	DMFS	87	1.190(0.840 , 1.700)	0.330
	GSE9195	DMFS	77	0.830(0.360 , 1.940)	0.670
	GSE9195	RFS	77	1.180(0.610 , 2.290)	0.630
	GSE12093	DMFS	136	1.050(0.600 , 1.850)	0.870
	GSE11121	DMFS	200	0.670(0.410 , 1.100)	0.120
	GSE1378	RFS	60	1.080(0.780 , 1.490)	0.660
	GSE1379	RFS	60	1.160(0.790 , 1.700)	0.450
	GSE2034	DMFS	286	1.100 (0.840 ,1.440)	0.480
	GSE1456-GPL96	OS	159	1.030(0.650 , 1.640)	0.900
	GSE1456-GPL96	RFS	159	0.780(0.480 , 1.250)	0.300
	GSE1456-GPL96	DSS	159	0.990(0.570 , 1.720)	0.990
	GSE7378	DFS	54	0.630(0.250 , 1.570)	0.320
	E-TABM-158	DMFS	117	1.160(0.860 , 1.570)	0.320
	E-TABM-158	OS	117	1.130(0.860 , 1.570)	0.520
	E-TABM-158	RFS	117	1.160(0.860 , 1.570)	0.320
	E-TABM-158	DSS	117	1.130(0.780 , 1.620)	0.530
	GSE3494-GPL96	DSS	236	1.090(0.680 , 1.740)	0.720
	GSE4922-GPL96	DFS	249	0.930(0.650 , 1.330)	0.690
	GSE2990	DMFS	125	0.450(0.170 , 1.200)	0.110
	GSE2990	RFS	125	0.570(0.300 , 1.110)	0.100
	GSE2990	DMFS	54	1.060(0.660 , 1.710)	0.800
	GSE2990	RFS	62	1.080(0.740 , 1.580)	0.690
	GSE7390	RFS	198	1.220(0.910 , 1.640)	0.180
	GSE7390	DMFS	198	1.050(0.730 , 1.510)	0.780
	GSE7390	OS	198	1.010(0.700 , 1.450)	0.970
Colorectal cancer	GSE12945	OS	62	5.240(1.360 , 20.240)	0.020
	GSE17536	DSS	177	1.040(0.360 , 3.010)	0.940
	GSE17536	OS	177	0.780(0.310 , 1.940)	0.590
	GSE17536	DFS	145	1.980(0.540 , 7.330)	0.310
	GSE14333	DFS	226	1.620(0.820 , 3.200)	0.160
	GSE17537	OS	55	0.320(0.070 , 1.400)	0.130
	GSE17537	DFS	55	0.550(0.110 , 2.860)	0.480
	GSE17537	DSS	49	0.180(0.030 , 1.120)	0.070
Head and neck cancer	GSE2837	RFS	28	2.140(1.040 , 4.400)	0.040
Lung cancer	jacob-00182-CANDF	OS	82	1.110(0.660 , 1.890)	0.690
	jacob-00182-HLM	OS	79	1.600(1.080 , 2.380)	0.020
	jacob-00182-MSK	OS	104	0.970(0.620 , 1.530)	0.900
	GSE13213	OS	117	1.240(0.870 , 1.760)	0.240
	GSE31210	OS	204	4.700(2.360 , 9.360)	<0.001
	GSE31210	RFS	204	3.440(2.030 , 5.810)	<0.001
	jacob-00182-UMI	OS	178	1.290(0.910 , 1.830)	0.150
	GSE11117	OS	41	1.990(1.220 , 3.230)	<0.001
	GSE3141	OS	111	1.090(0.790 , 1.490)	0.600
	GSE14814	OS	90	0.680(0.260 , 1.740)	0.420
	GSE14814	DSS	90	0.940(0.430 , 2.080)	0.880
	GSE8894	RFS	138	1.040(0.850 , 1.280)	0.690
	GSE4573	OS	129	1.110(0.730 , 1.700)	0.610
	GSE17710	OS	56	1.130(0.660 , 1.920)	0.660
	GSE17710	RFS	56	0.940(0.600 , 1.490)	0.800
	GSE17710	RFS	56	1.000(0.650 , 1.530)	1.000
	GSE17710	OS	56	1.010(0.630 , 1.600)	0.980
	GSE17710	RFS	56	1.030(0.630 , 1.700)	0.900
	GSE17710	OS	56	1.070(0.680 , 1.690)	0.760
Ovarian cancer	GSE9891	OS	278	0.820(0.560 , 1.190)	0.290
	DUKE-OC	OS	133	1.040(0.800 , 1.350)	0.780
	GSE8841	OS	81	1.260(0.450 , 3.520)	0.650
	GSE26712	DFS	185	1.580(1.000 , 2.490)	0.050
	GSE26712	OS	185	1.930(1.180 , 3.140)	<0.001
	GSE17260	PFS	110	1.040(0.750 , 1.440)	0.820
	GSE17260	OS	110	1.190(0.790 , 1.800)	0.410
	GSE14764	OS	80	0.720(0.440 , 1.190)	0.210
Renal cell carcinoma	E-DKFZ-1F	OS	59	3.610(0.960 , 13.610)	0.060
Skin cancer	GSE19234	OS	38	1.720(0.760 , 3.900)	0.190
Soft tissue cancer	GSE30929	DRFS	140	0.430(0.220 , 0.820)	0.010

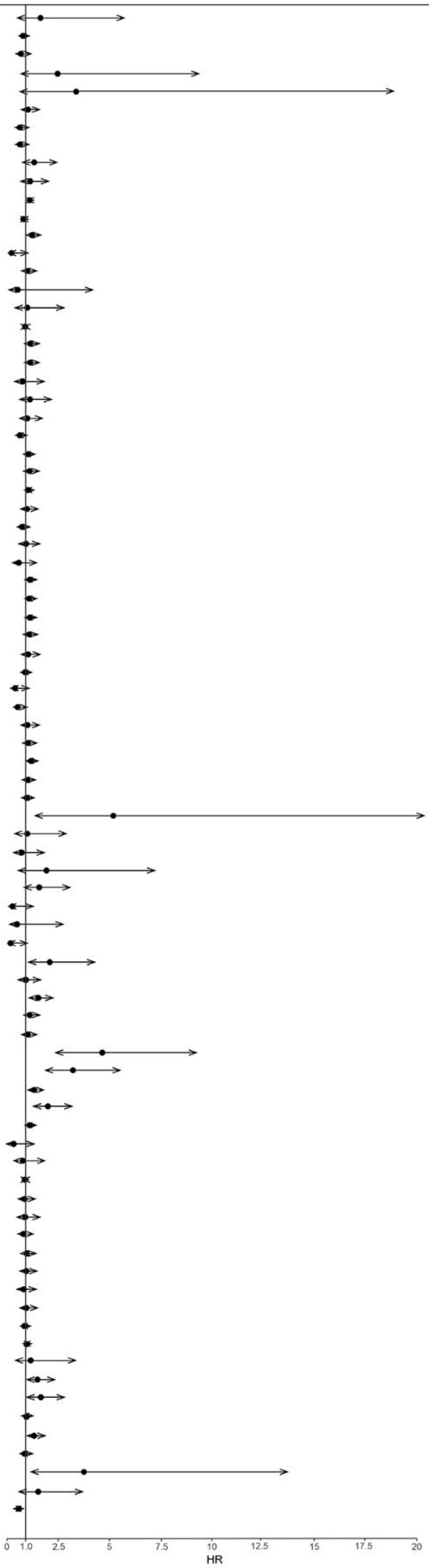

Supplementary Table 3. Associations between TSKU methylation and prognosis of different cancers in the methSurv database.

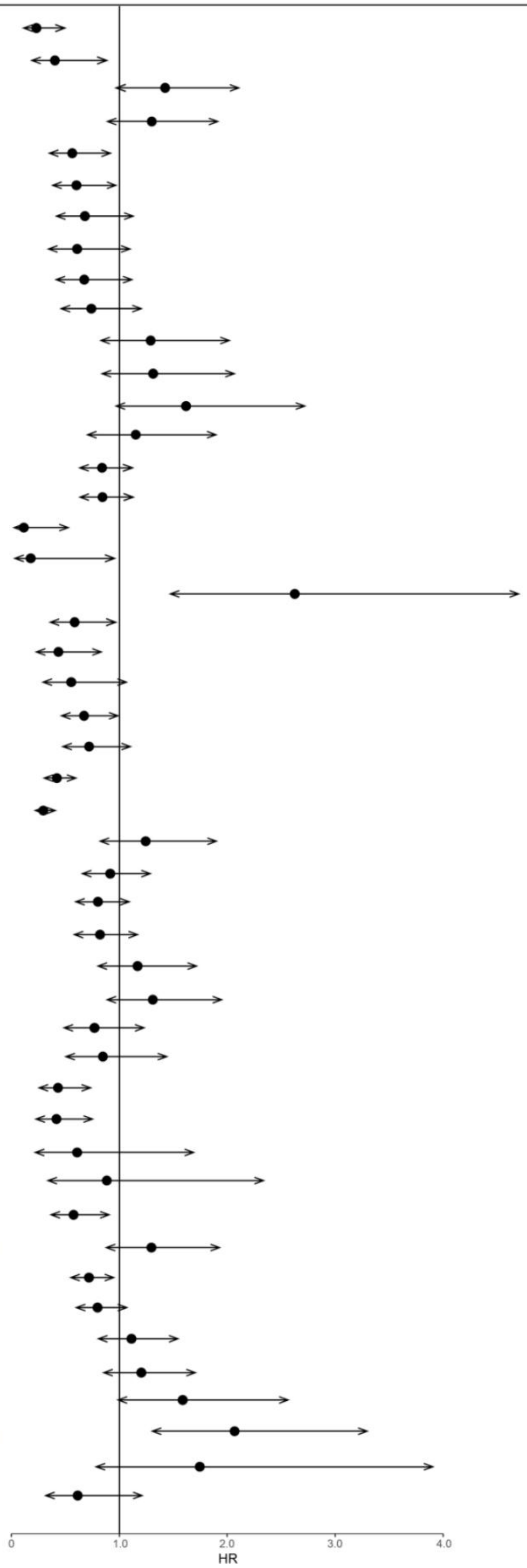

Cancer type	Promoter probe	Endpoint	N	Hazard ratio(95%CI)	*P*-value
ACC	cg20708135	OS	80	0.259(0.124 , 0.541)	<0.001
	cg20886049	OS	80	0.431(0.200 , 0.929)	0.032
BLCA	cg20708135	OS	412	1.452(0.980 , 2.152)	0.063
	cg20886049	OS	412	1.328(0.901 , 1.957)	0.150
BRCA	cg20708135	OS	782	0.594(0.366 , 0.965)	0.035
	cg20886049	OS	782	0.629(0.392 , 1.011)	0.056
CESC	cg20708135	OS	307	0.709(0.429 , 1.172)	0.180
	cg20886049	OS	307	0.638(0.356 , 1.144)	0.130
COAD	cg20708135	OS	293	0.703(0.425 , 1.161)	0.170
	cg20886049	OS	293	0.772(0.476 , 1.251)	0.290
ESCA	cg20708135	OS	185	1.319(0.842 , 2.064)	0.230
	cg20886049	OS	185	1.347(0.858 , 2.116)	0.200
GBM	cg20708135	OS	139	1.646(0.981 , 2.763)	0.059
	cg20886049	OS	139	1.180(0.717 , 1.940)	0.520
HNSC	cg20708135	OS	527	0.866(0.643 , 1.166)	0.340
	cg20886049	OS	527	0.869(0.644 , 1.172)	0.360
KICH	cg20708135	OS	66	0.143(0.036 , 0.571)	0.006
	cg20886049	OS	66	0.207(0.043 , 1.001)	0.050
KIRC	cg20708135	OS	319	2.653(1.484 , 4.744)	0.001
	cg20886049	OS	319	0.616(0.373 , 1.015)	0.060
KIRP	cg20708135	OS	275	0.473(0.252 , 0.887)	0.020
	cg20886049	OS	275	0.588(0.310 , 1.116)	0.100
LAML	cg20708135	OS	194	0.707(0.481 , 1.038)	0.077
	cg20886049	OS	194	0.741(0.481 , 1.141)	0.170
LGG	cg20708135	OS	515	0.439(0.305 , 0.632)	<0.001
	cg20886049	OS	515	0.301(0.210 , 0.429)	<0.001
LIHC	cg20708135	OS	377	1.272(0.833 , 1.944)	0.270
	cg20886049	OS	377	0.948(0.673 , 1.336)	0.760
LUAD	cg20708135	OS	461	0.829(0.606 , 1.136)	0.240
	cg20886049	OS	461	0.866(0.610 , 1.230)	0.420
LUSC	cg20708135	OS	372	1.198(0.815 , 1.761)	0.360
	cg20886049	OS	372	1.338(0.898 , 1.993)	0.150
MESO	cg20708135	OS	87	0.792(0.494 , 1.268)	0.330
	cg20886049	OS	87	0.877(0.518 , 1.486)	0.630
PAAD	cg20708135	OS	184	0.483(0.291 , 0.802)	0.005
	cg20886049	OS	184	0.509(0.302 , 0.860)	0.012
READ	cg20708135	OS	97	0.641(0.236 , 1.737)	0.380
	cg20886049	OS	97	0.909(0.347 , 2.378)	0.850
SARC	cg20708135	OS	261	0.644(0.419 , 0.990)	0.045
	cg20886049	OS	261	1.325(0.890 , 1.973)	0.170
SKCM	cg20708135	OS	462	0.728(0.544 , 0.974)	0.033
	cg20886049	OS	462	0.819(0.606 , 1.106)	0.190
STAD	cg20708135	OS	395	1.176(0.853 , 1.623)	0.320
	cg20886049	OS	395	1.228(0.863 , 1.745)	0.250
UCEC	cg20708135	OS	431	1.618(1.003 , 2.611)	0.049
	cg20886049	OS	431	2.087(1.308 , 3.332)	0.002
UCS	cg20708135	OS	57	1.773(0.796 , 3.949)	0.160
	cg20886049	OS	57	0.645(0.331 , 1.258)	0.200
